# Anion Effect in Electrochemical CO_2_ Reduction: From Spectators to Orchestrators

**DOI:** 10.1021/jacs.4c10661

**Published:** 2024-10-15

**Authors:** Ji Mun Yoo, Johannes Ingenmey, Mathieu Salanne, Maria R. Lukatskaya

**Affiliations:** † Electrochemical Energy Systems Laboratory, Department of Mechanical and Process Engineering, ETH Zurich, 8092 Zurich, Switzerland; ‡ CNRS, Physicochimie des Électrolytes et Nanosystèmes Interfaciaux, Sorbonne Université, F-75005 Paris, France; § Institut Universitaire de France (IUF), 75231 Paris, France

## Abstract

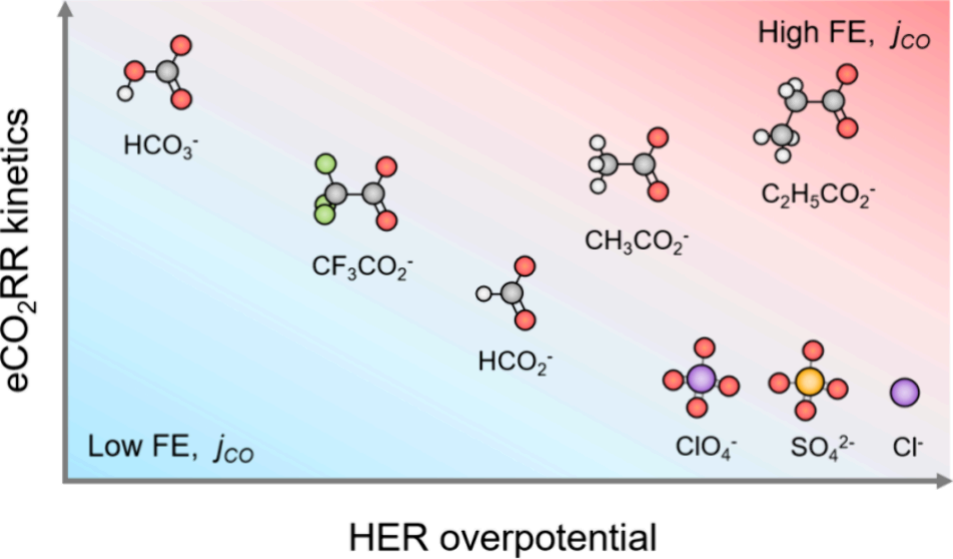

The electrochemical CO_2_ reduction reaction (eCO_2_RR) offers a pathway to produce valuable chemical fuels from CO_2_. However, its efficiency in aqueous electrolytes is hindered by the concurrent H_2_ evolution reaction (HER), which takes place at similar potentials. While the influence of cations on this process has been extensively studied, the influence of anions remains largely unexplored. In this work, we study how eCO_2_RR selectivity and activity on a gold catalyst are affected by a wide range of inorganic and carboxylate anions. We utilize *in situ* differential electrochemical mass spectrometry (DEMS) for real-time product monitoring coupled with molecular dynamics (MD) simulations. We show that anions significantly impact eCO_2_RR kinetics and eCO_2_RR selectivity. MD simulations reveal a new descriptor—free energy of anion physisorption—where weakly adsorbing anions enable favorable CO_2_ reduction kinetics, despite the negative charge carried by the electrode surface. By leveraging these fundamental insights, we identify propionate as the most promising anion, achieving nearly 100% Faradaic efficiency while showing high CO production rates that are comparable to those in bicarbonate. These insights underscore the vital role of anion selection in achieving a highly efficient eCO_2_RR in aqueous electrolytes.

## Introduction

Intense research efforts aimed at converting CO_2_, a greenhouse gas, into value-added chemicals and fuels have gained momentum due to growing concerns about environmental sustainability.^1,2^ The electrochemical CO_2_ reduction reaction (eCO_2_RR) has emerged as a promising approach, offering an avenue for sustainable carbon feedstock using renewable electricity at ambient conditions.^3−5^ However, eCO_2_RR in aqueous electrolytes has low Faradaic efficiency due to the concomitant H_2_ evolution reaction (HER) at the cathode surface that takes place at similar potentials. Since both reactions involve charge transfer at the electrified surface and deprotonation of water molecules, the selectivity of the process is largely determined by the local chemical environment at the electrochemical interface, including double-layer speciation, local pH, electric field modulation, and mass transport within the diffusion layer.^6−12^ Therefore, to maximize CO_2_ conversion efficiency, it is essential to understand the role of each electrolyte species (e.g., cations, anions, and solvent molecules) during the eCO_2_RR.

In this regard, current research efforts focus on formulating the design rules of electrolyte engineering for selective eCO_2_RR.^8,13−15^ For example, recent studies have revealed the critical role of alkali cation presence^16^ and its type (e.g., Li^+^, Na^+^, K^+^, and Cs^+^)^17−20^ and concentration^21^ in promoting eCO_2_RR selectivity. Big cations such as Cs^+^ and K^+^ show enhanced CO_2_ reduction activity by stabilizing the key reaction intermediates (i.e., adsorbed CO_2_^–^) through either a larger electric field^18^ or stronger electrostatic interactions^16^ compared to alkali cations with larger hydrated radii, such as Li^+^ and Na^+^. Also, the blocking of HER-active sites by cations was recently proposed by Qin et al. as a mechanism for HER suppression.^22^

In addition to the cation effect, anions can also play an important role in determining eCO_2_RR selectivity. However, the current understanding of the anion effect remains limited. Although it may seem counterintuitive that anions could play a crucial role given the negative charge of the cathode surface, which should theoretically repel them, our current understanding of the anion adsorption mechanism is limited. The interaction among various intermolecular forces can result in complex interfacial structures, indicating that anions might impact the reaction outcomes in unexpected ways. Bicarbonate (HCO_3_^–^) is widely employed as a benchmark anion in eCO_2_RR studies.^23−26^ Because of its chemical equilibrium with dissolved CO_2_, bicarbonate is postulated to increase the local CO_2_ concentration at the electrochemical interface, hence enhancing CO_2_ reduction kinetics.^23,24^ Moreover, bicarbonate as a buffer can suppress local pH increases near the catalyst surface, where hydroxide (OH^–^) ions are generated during catalytic reduction reactions. Because a local pH increase from hydroxide generation can shift the chemical equilibrium between CO_2_ and bicarbonate toward less CO_2_ (i.e., CO_2_ depletion) at the catalytic interface, buffer anion-like bicarbonate can facilitate a higher CO_2_ concentration at the catalytic interface compared to a nonbuffered system. Lee and co-workers studied eCO_2_RR selectivity in the presence of chloride, sulfate, and phosphate anions and compared it to the bicarbonate case on a Au catalyst at a fixed potential of *E* = −0.7 V (vs RHE).^27^ It was suggested that chloride improves CO selectivity compared to bicarbonate by suppressing HER through specific adsorption on Au. Similarly, in the case of the Cu catalyst, it was shown that halide anions (Cl^–^, Br^–^, and I^–^) have a significant impact on eCO_2_RR selectivity.^28−30^ As these ions exhibit strong adsorption on the metal surfaces,^28,29^ they can partially block or change the electronic structure of the active sites and facilitate or suppress specific reaction pathways.^30,31^ Moreover, strong adsorption of halide anions can induce Cu surface faceting through electrochemical roughening, leading to the development of eCO_2_RR-active crystal facets.^31,32^ Still, compared to the well-established cationic effect on eCO_2_RR selectivity, the systematic investigation of the anion effect has been limited, with few studies focusing on the anion role with the Cu catalyst^28−32^ and a single study for Au catalysts.^27^ Therefore, a proper understanding of how different anions influence the eCO_2_RR process is still lacking, hindering the development of the design rules for achieving highly selective CO_2_ conversion efficiency.

To address this, we systematically studied the effect of anions on eCO_2_RR activity and selectivity on the Au catalyst. We explored various electrolytes containing anions such as bicarbonate, perchlorate, sulfate, chloride, and carboxylates, and potassium as a cation (due to its eCO_2_RR-promoting role^16,18^). We also studied the influence of carboxylate anions due to their structural closeness to bicarbonate. We selected acetate (CH_3_COO^–^), propionate (C_2_H_5_COO^–^), formate (HCOO^–^), and trifluoroacetate (CF_3_COO^–^) as representative cases with both electron-withdrawing and electron-donating groups. We employed *in situ* differential electrochemical mass spectrometry (DEMS)^20,33^ for monitoring the production rates of both H_2_ and CO molecules on the Au catalyst surface as a function of potentials and selected anions. Furthermore, since all testing was performed under identical cell configuration conditions in our work, we were able to generate a systematic 1-to-1 comparison across a wide range of anions.

## Results and Discussion

### The Effect of Noncarboxylate Anions

**Figure 1:**
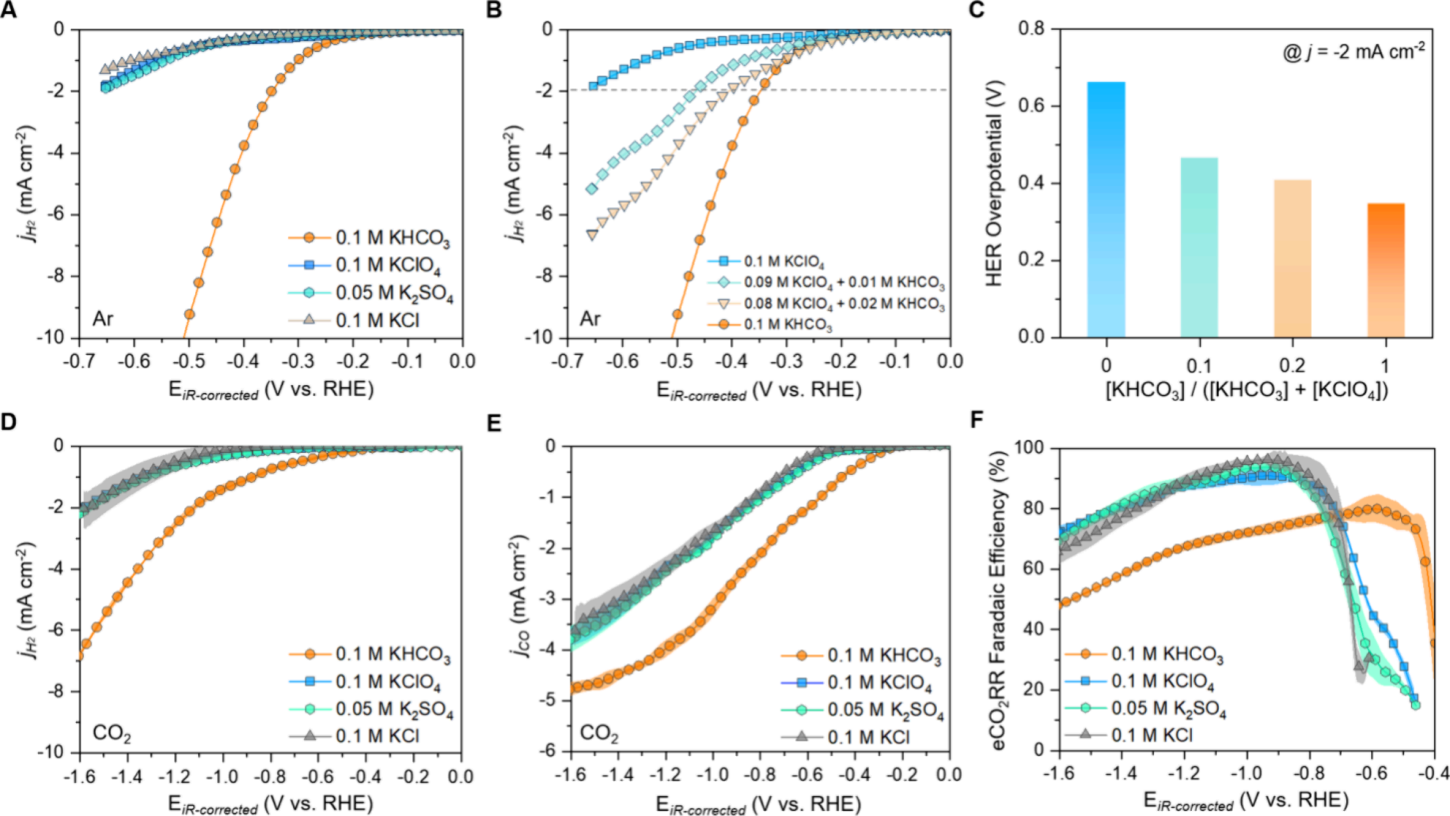
(a, b) HER activity of the polycrystalline Au electrode using a rotating disk electrode in Ar-saturated K^+^-based electrolytes: (a) bicarbonate, perchlorate, sulfate, and chloride anions and (b) mixture of bicarbonate and perchlorate anions (scan rate = 5 mV s^–1^, rotation speed = 3000 rpm), and (c) HER overpotential at the current density (*j*_H2_) of −2 mA cm^–2^. (d) HER and (e) eCO_2_RR partial current density on the Au thin-film catalyst in CO_2_-saturated electrolytes (scan rate = 5 mV s^–1^, flow rate = 60 mL min^–1^). (f) Faradaic efficiency for eCO_2_RR in different electrolytes. The translucent area represents the error bar at each potential calculated from the standard deviation of three individual measurements.

First, we evaluate the anion effect on HER kinetics in the absence of CO_2_ on polycrystalline Au using rotating disk electrode (RDE) measurements for the perchlorate, sulfate, and chloride anions and benchmark them against bicarbonate. In all cases, the potassium (K^+^) cation concentration was fixed at 0.1 M.^16,18^ Similar HER current densities were observed for all anions except bicarbonate, which showed much higher HER activity (Figure 1a). This correlates well with previous reports by Resasco et al.^34^ and Marcandalli et al.^14^ where bicarbonate promoted HER. Such a high HER in the bicarbonate electrolyte can be explained by its p*K*_a_ value being lower than that of water (p*K*_a,HCO3–_ = 10.3 and p*K*_a,H2O_ = 14) and therefore having a more favorable proton-donating ability compared to water molecules. We further corroborated the bicarbonate-driven HER by performing additional experiments in perchlorate–bicarbonate mixtures at different ratios while maintaining [K^+^] = 0.1 M (Figure 1b). When the bicarbonate molar fraction increases, lower HER overpotentials are observed (Figure 1c).

Next, we quantify the respective eCO_2_RR and HER contributions in CO_2_-saturated electrolytes using *in situ* DEMS analysis (Figures S1–S9; see the Experimental Section for details). CO is found to be a major eCO_2_RR product on the Au catalyst (Figure S7). We found that the HER activity trend was similar to the Ar-saturated electrolyte case: HER currents were notably higher in the bicarbonate electrolyte compared to the other anions (Figure 1d). Meanwhile, the CO_2_-to-CO conversion has an earlier onset potential in the electrolyte containing bicarbonate (−0.3 V vs RHE) compared to the other anions (−0.5 V vs RHE) (Figure 1e). An earlier eCO_2_RR onset can be also observed in the case of bicarbonate when eCO_2_RR activity is compared on the Standard Hydrogen Electrode (SHE)-scale (Figure S10). These results can be rationalized by an increase in the transient CO_2_ concentration near the catalyst surface by bicarbonate, as suggested by Dunwell et al.^23^ and Zhu et al.^24^ Meanwhile, we observe negligible differences in eCO_2_RR and HER activities for perchlorate, sulfate, and chloride anions.

Next, we compare the Faradaic efficiency (FE) for the CO_2_-to-CO conversion as a function of applied potential and anion type (Figure 1f). Bicarbonate features the well-known bell curve where its peak value of 80% appears at −0.6 V (vs RHE), corroborating the previous reports^35−37^: first, FE rapidly increases as CO evolution starts at −0.4 V, then it peaks at around 80% (−0.6 V) and decreases below −0.6 V. Although other anions show much lower HER partial current densities compared to bicarbonate, they suffer from lower selectivity at >−0.8 V due to poor eCO_2_RR kinetics. However, at potentials below the eCO_2_RR onset of *E* ≈ −0.5 V, FE increases rapidly, reaching peak values of over 90%, which is notably higher than with bicarbonate (Figure 1f). For all electrolytes, once the FE peak is reached, the selectivity decreases at more negative potentials due to an increased contribution of HER from water molecules. Our results show that bicarbonate anions can negatively affect reaction selectivity, despite facilitating notably higher rates of CO production. In contrast, anions like perchlorate, sulfate, and chloride can enable high FE of CO production, however showing slower eCO_2_RR rates.

### Effect of Carboxylate Anions

Carboxylate anions have a negatively charged carboxyl group and, therefore, are structurally similar to bicarbonate anions. Therefore, they can potentially promote eCO_2_RR similar to bicarbonate, yet so far there have been almost no reports studying carboxylate-based electrolytes for eCO_2_RR in aqueous media. Here, we study the eCO_2_RR activity and selectivity in acetate, propionate, formate, and trifluoroacetate electrolytes. Each of these anions has a different electron density on the carboxylate group and, therefore, has a distinct p*K*_a_ value (Table S1).

**Figure 2:**
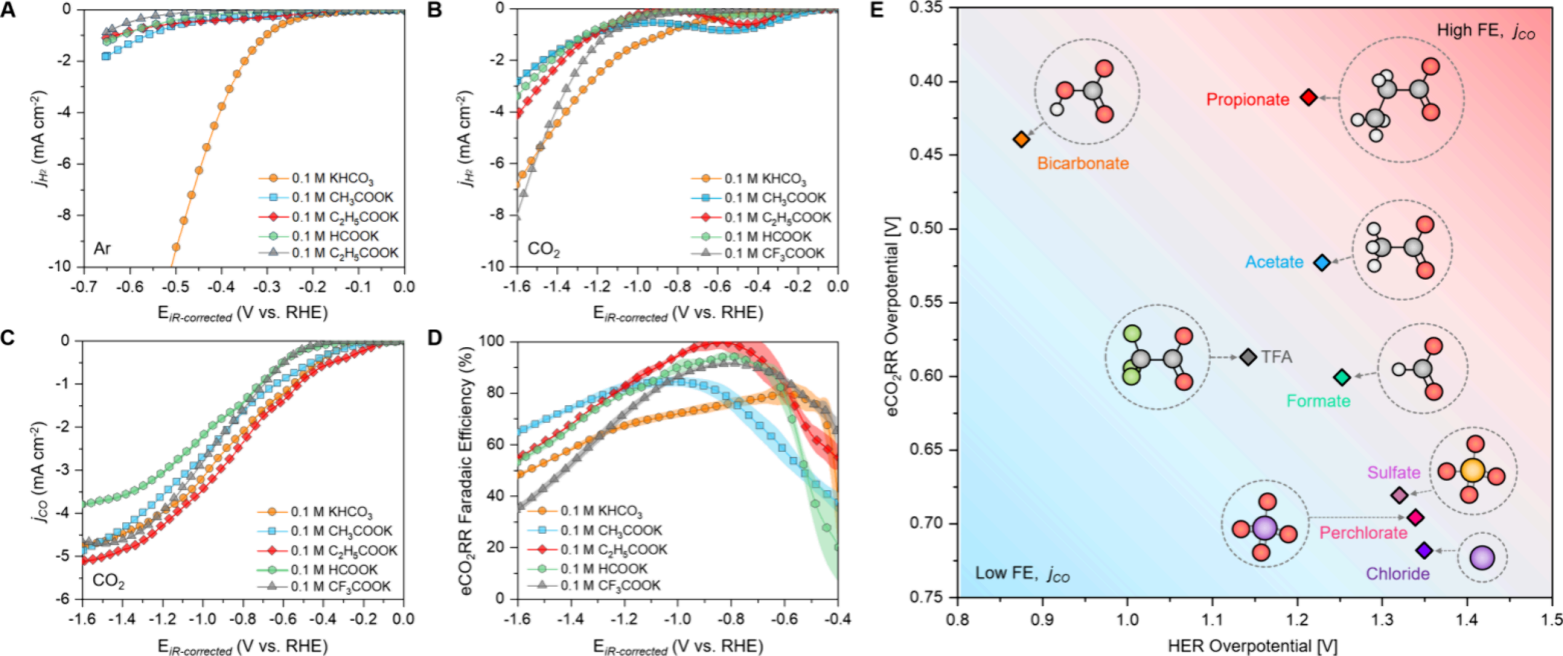
(a) HER activity of the polycrystalline Au electrode using a rotating disk electrode (scan rate = 5 mV s^–1^, rotation speed = 3000 rpm). (b) HER (*j*_H2_) and (c) eCO_2_RR (*j*_CO_) partial current densities on the Au thin-film catalyst in CO_2_-saturated electrolytes (scan rate = 5 mV s^–1^, flow rate = 60 mL min^–1^) (d) Faradaic efficiency for the eCO_2_RR in each carboxylate electrolyte. The translucent area represents error bars at each potential calculated as the standard deviation of three individual measurements. (e) Overpotential for HER and eCO_2_RR at a partial current density of *j* = −1 mA cm^–2^ for electrolytes with different anions.

First, we analyze the HER activity in Ar-saturated electrolytes. All carboxylate anions show a higher overpotential for HER compared to bicarbonate (Figure 2a). This is due to the higher proton-donation ability of bicarbonate compared to water which leads to bicarbonate-driven HER kinetics in addition to water-driven HER.^14,34^ On the other hand, when electrolytes are saturated with CO_2_, additional partial HER currents emerge at potentials between −0.1 and −0.7 V (vs RHE) in carboxylate-based electrolytes (*in situ* DEMS results: Figure 2b). Considering the lower pH value of CO_2_-saturated electrolytes compared to Ar-saturated ones (Table S2) and the p*K*_b_ values of these anions (Table S1), these additional HER currents can be attributed to HER from conjugate acids (e.g., CH_3_COOH and C_2_H_5_COOH) since their equilibrium concentration becomes non-negligible at lower pH due to CO_2_ saturation. For instance, 8.4 mM of CH_3_COOH is estimated to be present as the conjugate acid in the CO_2_-saturated 0.1 M CH_3_COOK electrolyte (pH = 5.8, p*K*_a_ = 4.75). When we adjust the pH of Ar-saturated 0.1 M CH_3_COOK to 5.8 by adding 8.4 mM CH_3_COOH, the HER activity on the Au surface shows the same onset of HER current at −0.1 V, as shown in Figure 2b (see Figure S11 for the details). However, when the potential is lowered further, the HER current density decreases and proceeds through a minimum before rising again. We suggest that an increase in local pH decreases the concentration of the conjugate acid within the diffusion layer. Similar assumptions are valid for the propionate case. This HER feature is nearly absent in formate and completely disappears in trifluoroacetate electrolytes due to a negligible concentration of conjugate acid molecules expected due to their high p*K*_b_ (Table S1).

Next, we analyze the effect of carboxylate anions on eCO_2_RR partial currents and CO evolution onsets (Figure 2c). In contrast to noncarboxylate anions that are characterized by more sluggish eCO_2_RR kinetics (i.e., eCO_2_RR onsets of −0.5 V), both acetate and propionate anions reveal an eCO_2_RR onset potential at −0.2 V and eCO_2_RR current density closely comparable to bicarbonate electrolytes. Meanwhile, the eCO_2_RR in 0.1 M KHCOO and 0.1 M KCF_3_COO electrolytes is characterized by higher overpotentials compared to bicarbonate (Table S3).

Finally, Figure 2d shows anion-specific trends in the FE as a function of applied potential. At low overpotentials (−0.4 to −0.6 V), we observe low overall current densities (Figure S12) and lower Faradaic efficiency for CO production for acetate and propionate anions than bicarbonate due to larger HER currents from the conjugate acids. For the intermediate potential range (−0.8 to −0.6 V), a much higher FE can be achieved in all carboxylate electrolytes compared to bicarbonate. The highest FE of 98.7% (±1.3%) is observed at −0.8 V for propionate. Formate shows the FE = 94.2% (±1.7%), and trifluoroacetate shows the FE = 91.5% (±0.5%). The lowest FE (84 ± 1.0%) is reached in acetate electrolytes due to a significant contribution of acetic acid-driven HER (Figure 2b). Our results show that some carboxylate anions can enable significantly higher eCO_2_RR selectivities than bicarbonate and present a promising electrolyte system to minimize the HER at high eCO_2_RR current conditions.

Figure 2e summarizes the observed anion dependence of HER and eCO_2_RR activity by depicting their respective overpotentials at a partial current density of *j* = −1 mA cm^–2^ (note: partial current density is selected low enough to minimize the possible effect from mass transfer limitation). Bicarbonate demonstrates favorable CO_2_ reduction kinetics (i.e., low eCO_2_RR overpotential) but exhibits diminished product selectivity due to its concurrently low HER overpotential. For perchlorate, sulfate, or chloride anions, a large HER overpotential enables a selectivity of over 80%; however, the eCO_2_RR kinetics is poor and characterized by a high eCO_2_RR overpotential. Finally, carboxylate anions effectively suppress HER (due to large HER overpotentials) while enabling high eCO_2_RR activity. The respective selectivity of eCO_2_RR varies depending on the carboxylate type, reaching a maximum of nearly 100% in the case of propionate, offering a simple alternative to bicarbonate or conventional inorganic anions.

It is important to note that, when comparing the influence of different anions on eCO_2_RR selectivity, we should also consider the impact of local pH changes and potential bicarbonate formation. The studied anions, except for bicarbonate, have low buffer capacity, which can lead to a local pH increase at the electrocatalytic interface, affecting HER kinetics. To assess this, we used *in situ* DEMS analysis to monitor local pH changes by comparing *m*/*z* signals for consumed CO_2_ and evolved CO (Figure S13). A divergence between these signals indicates a local pH increase since the dissolved CO_2_ concentration is highly sensitive to pH (due to chemical CO_2_ depletion through its equilibrium with bicarbonate at increased pH).^33^ We performed this analysis for three representative electrolytes: bicarbonate, propionate, and perchlorate. For 0.1 M KHCO_3_, the signals matched closely for the studied current density range, indicating a well-buffered system with no significant local pH change (Figure S13a). In the case of 0.1 M C_2_H_5_COOK and 0.1 M KClO_4_ (Figure S13b,c), the two mass signals remained closely matched down to *E* = −1.0 V (vs RHE). Below this potential, CO_2_ consumption increases faster than CO evolution, indicating CO_2_ depletion due to the local pH increase at the interface in addition to CO_2_-to-CO electrochemical conversion. The low buffer capacity of propionate and perchlorate anions can rationalize this result. However, it should be noted that no significant local pH change was observed down to *E* = −1.0 V (vs RHE), corresponding to *j*_CO_ = −3.5 mA cm^–2^ for 0.1 M C_2_H_5_COOK. Therefore, as the activity comparison in Figure 2e is presented for lower current densities of −1 mA cm^–2^, the influence of local pH change can be considered negligible for these conditions.

Next, we also considered unintended bicarbonate formation due to CO_2_ hydration and its chemical equilibrium (see the detailed discussion in the Supporting Information). CO_2_ solubility is similar across electrolytes (Figure S14), and the respective bicarbonate concentration calculations are summarized in Figure S15 and Table S4. In the case of formate, trifluoroacetate, perchlorate, sulfate, and chloride, bicarbonate formation is largely limited (<0.001 M) due to the low pH value of the electrolytes after CO_2_ saturation (Table S4), so its effect on electrocatalytic response should be largely limited. For acetate and propionate, the estimated bicarbonate concentration is ∼10 mM; therefore, it could potentially influence the observed HER currents.^14,34^ To study this, we performed an RDE experiment in a 0.09 M CH_3_COOK + 0.01 M KHCO_3_ electrolyte. Only a marginal HER activity increase was observed (Figure S16), thus indicating that bicarbonate presence in acetate/propionate does not significantly affect HER activity. Instead, we found out that the increased HER activity arises from the conjugate acid (e.g., CH_3_COOH for acetate, see Figure S11). Thus, we suggest that the anion-dependent activity and selectivity trends shown in Figure 2e can be attributed directly to the anions studied.

### Molecular Dynamics Simulations

To further understand the anion-dependent eCO_2_RR activity, a series of molecular dynamics (MD) simulations of the gold/electrolyte interfaces were conducted for representative cases of bicarbonate, propionate, trifluoroacetate, and perchlorate anions. This approach allows us to assess the effects of different electrolyte chemistry on the electrocatalytic performance due to variations in the electrochemical double-layer structure. The systems were simulated following previous works on similar systems by putting the liquid electrolytes in contact with two Au(111) electrodes under constant applied potentials between them (see the simulation setup in Figure S17).^38,39^ Within the constant potential method, although it is not possible to fix each electrode potential as in experiments, the partial charges of the electrode atoms are allowed to fluctuate with time. For example, an average negative charge of −3.5 μC/cm^2^ is obtained for the cathode at an applied potential of 1 V (fluctuations with time of the total electrode charge are shown in Figure S18). Analysis of the equilibrium simulations reveals that regardless of the surface charge, anions display a strong affinity to the surface. This behavior results from an interplay between electrostatic effects and other interactions. Since anions are less strongly solvated than cations, they prefer to sit close to the electrode interface, even when it carries a negative total charge. Indeed, visual analysis of the local excess charge on the anion adsorption sites (Figure S19) shows that the corresponding gold atoms adopt a slightly positive charge. In contrast, the potassium cations do not adsorb directly onto the electrode surface. Instead, they are positioned between the two first water layers, enabling them to maintain their solvation shells largely intact. It is worth noting that the adsorption profile of the K^+^ cations is not influenced by the nature of the anions, further highlighting their preferential interaction with the water molecules.

**Figure 3:**
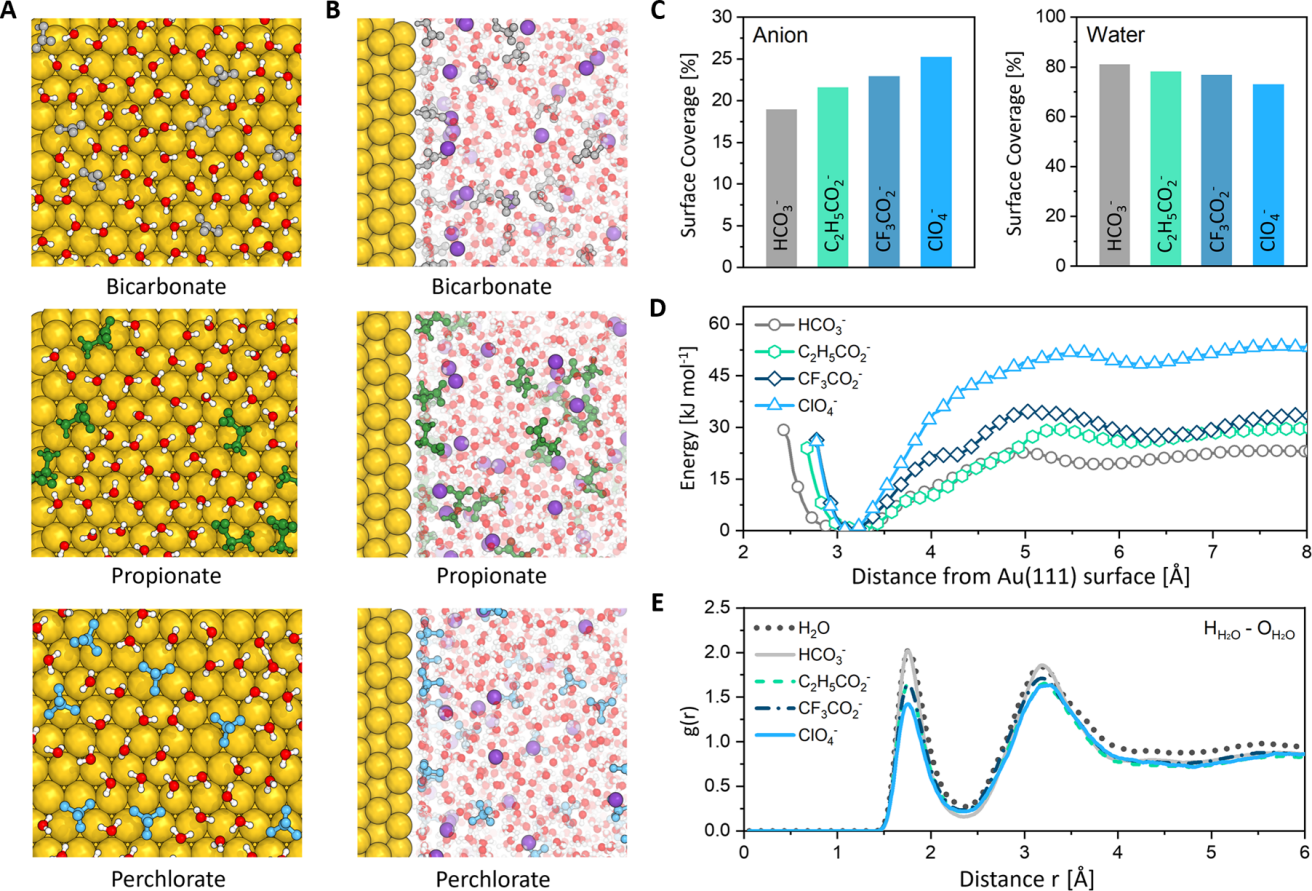
Molecular dynamics calculation: visualized snapshot image of (a) Au(111) surface and (b) Au(111)–electrolyte interface. (c) Calculated surface coverage of anion and water molecules on the Au(111) cathode surface. (d) Potential of mean force (PMF) and (e) radial distribution function (RDF) of H_H_2_O_–O_H_2_O_ distance within the Au(111) cathode−electrolyte interface for different electrolytes.

When the anions are compared, some differences appear, with perchlorate anions preferentially adsorbing onto the Au(111) surface compared to bicarbonate and the two carboxylate anions (i.e., propionate and trifluoroacetate). This preferential adsorption is evident from the higher population of the perchlorate anions on the Au surface in the MD snapshots compared to bicarbonate and propionate (Figure 3a–c). Furthermore, the calculated surface coverage shows that perchlorate occupies a larger portion of the Au(111) surface compared with the other two anions (Figure 3c and Table S5). The strongly adsorbed anions partially displace water molecules on the Au surface, an effect that is much less important in the case of carboxylate anions. The equilibrium distance is also different between the three species: the peak density for perchlorate is located notably closer to the Au(111) surface (3.16 Å) compared to bicarbonate and propionate (3.3 Å), an effect that can be attributed to the relative size of the molecules (Figure S20).

Next, to get more quantitative results, constrained simulations were performed at 0 V (Figure 3d) wherein one anion is progressively dragged toward the surface. This allowed us to extract the free energy profile for the physisorption of the ions, which measures the free energy variations between the bulk liquid and the surface. This quantity correlates well with the results from equilibrium simulations (discussed below). First, for all studied anions, there is no free energy barrier to reach the second adsorbed layer (located at ∼6 Å from the cathode surface, approximately) and a very small barrier (5 kJ mol^–1^ at most) for jumping from the second to the first adlayer (located at ∼3 Å), which supports that they will not encounter any kinetic hindrance for getting adsorbed. Second, the free energies of anion physisorption (defined as the free energy in the first layer minus the one in the bulk) differ significantly, with the following order: ClO_4_^–^ (−52 kJ mol^–1^) ≫ CF_3_COO^–^ (−35 kJ mol^–1^) > C_2_H_5_COO^–^ (−29 kJ mol^–1^) > HCO_3_^–^ (22 kJ mol^–1^). This allows us to differentiate them into two groups: strong physisorbing (perchlorate) and weak physisorbing (bicarbonate and propionate). This classification correlates well with the observed eCO_2_RR kinetics, as summarized in Figure 2e. Therefore, we propose that this free energy of anion physisorption can be used as a descriptor of the electrolyte performance for the CO_2_-to-CO electrocatalytic reaction on Au. Here, we note that the order between propionate and bicarbonate is reversed compared to the experiments; this discrepancy might be due to simulation approximations, such as the force field selection. However, the physisorption energy difference between them is very small compared to their difference to perchlorate; thus, we propose that the free energy of physisorption can be used to discriminate and predict electrolytes with favorable or unfavorable eCO_2_RR kinetics. In order to examine the effect of the electrode potential (and thus the negative electrode charge) on the potential of mean force, we also computed it for an applied potential of 1 V. As shown in Figure S21, the profiles remain very similar, although the minimum free energies are increased by approximately 5 kJ mol^–1^ compared to the 0 V case, due to the additional electrostatic repulsion. This result is consistent with our observation that anions show a strong tendency to adsorb on the electrode surface under all the studied conditions.

Concerning the HER overpotential, recent studies have suggested the importance of interfacial water reorganization, either from experiments^40^ or from MD simulations.^41^ Radial distribution functions (RDF) obtained for the molecules adsorbed within the first layer (Figure 3e) reveal a diminished water–water interaction in the following order: ClO_4_^–^ < CF_3_COO^–^ ≈ C_2_H_5_COO^–^ < HCO_3_^–^. Notably, the bicarbonate case closely resembles that of pure water (Figure S22). Therefore, we conclude that the presence of the anions strongly affects the hydrogen bonding network of interfacial water molecules. Such a structural change can have two possible implications. A weakened hydrogen bonding network can significantly impact HER kinetics by hindering the transfer of H^+^ and/or newly formed OH^–^ anions through a Grotthuss-like mechanism during HER, although it is difficult to get direct simulation proof.^42^ Alternatively, the local activity coefficients of the adsorbed water molecules could be altered, which could lead to a shift in the HER potential. In conclusion, because anions can block effective active sites on the catalyst surface and induce a change in the water network structure, we propose that the resulting selectivity (or FE) of a given electrolyte arises from the structure and energetics of the anion physisorption process in this studied series of electrolytes. In addition, as noted above, some anions (e.g., bicarbonate) can also contribute to the HER due to their acidic character, an effect that cannot be taken into account in our classical MD simulations.

## Conclusions

In summary, this study emphasizes the crucial role of anions in controlling HER and maximizing CO_2_ reduction, paving the way for the optimized electrolyte design for electrochemical CO_2_ conversion systems. We reveal the effect of the anion on the evolution of H_2_ and CO molecules during electrochemical CO_2_ reduction using *in situ* DEMS, covering a wide range of inorganic and carboxylate anions. We show that compared to bicarbonate, inorganic anions (i.e., perchlorate, sulfate, and chloride) suppress HER (due to the absence of bicarbonate-driven HER) and also lead to lower CO production rates and higher eCO_2_RR overpotentials. Next, by studying carboxylate anions with different molecular structures, we found that specific carboxylate electrolytes promote more favorable CO_2_ reduction kinetics compared to inorganic anions, with the propionate anion promoting the highest eCO_2_RR activity. Importantly, compared to bicarbonate, carboxylate anions offer significantly higher CO_2_ reduction selectivity by suppressing the HER without compromising the CO_2_ reduction rates. Peak Faradaic efficiency values demonstrate this trend: acetate (84%) < trifluoroacetate (91%) < formate (94%) < propionate (99%). Using MD simulations of the electrical double layer, we rationalize this anion dependence by correlating eCO_2_RR activity with the free energies of anion physisorption. We propose the physisorption energy of anions as a descriptor, with lower values enabling more favorable eCO_2_RR kinetics. Meanwhile, water–anion interactions can weaken the hydrogen bond network of water molecules in the first adsorbed layer and result in higher HER overpotentials.

## Experimental Methods

### Chemicals

Electrolytes were prepared from KHCO_3_ (99.7%, Sigma-Aldrich), CH_3_COOK (≥99.0%, Sigma-Aldrich), C_2_H_5_COOK (≥98%, TCI), HCOOK (99.9%, Sigma-Aldrich), CF_3_COOK (≥98%, TCI), KClO_4_ (≥99%, Sigma-Aldrich), K_2_SO_4_ (≥99%, Sigma-Aldrich), KCl (99.5%, Sigma-Aldrich), and ultrapure water (Milli-Q grade, ≥18.2 MΩ cm, ACCU 20, Scientific Fisher). H_2_SO_4_ (95.0–98.0%, ACS reagent, Sigma-Aldrich) was used for electrochemical cell cleaning. Ar (5.0 purity, PanGas) and CO_2_ (4.5 purity, PanGas) were used for purging the electrolytes.

### Electrolyte Purification

To remove metallic impurities, each electrolyte was electrochemically purified by applying a current density of 0.1 mA cm^–2^ between two Au electrodes for 12 h.^20,43^ HCOOK electrolytes were used without electrochemical purification to prevent formate oxidation reactions on the anode side.

### Electrochemical Measurements

All electrochemical measurements were conducted using a VSP-300 potentiostat (Biologic) equipped with EC-LAB software. The onset potential of HER and eCO_2_RR is defined as the potential value at −0.1 mA cm^–2^ of partial current density in this work because it corresponds to the DEMS detection limit threshold. For each experiment, *iR* correction was applied at 85% compensation of the uncompensated resistance measured by electrochemical impedance spectroscopy. Three independent experiments were conducted for each electrolyte for the calculation of error bars. All electrochemical potentials were converted from the Ag/AgCl scale to RHE using the following equation:
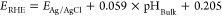


### RDE Experiment

RDE experiments were conducted in an H-type glass cell in a three-electrode configuration using RRDE-3A equipment (ALS). A polycrystalline Au disk (ALS) was used as the working electrode. A leakless Ag/AgCl electrode (eDAQ, 3.4 M KCl) and a large area graphite rod were used as the reference and counter electrodes, respectively. The Nafion 211 membrane (Sigma-Alrich) was used to separate the catholyte and anolyte to avoid product crossover. Before the RDE experiment, the Nafion membrane was cation-exchanged by immersing in 0.1 M NaOH and rinsing with copious amounts of DI water.

Prior to each experiment, the glass cell was cleaned by immersing in 0.05 M H_2_SO_4_ solution for 1 h, followed by boiling in DI water for 10 min, which was repeated two times. The Au electrode was polished with 0.3 and 0.05 μm alumina powders (CH Instrument) sequentially and ultrasonicated in a 1:1 mixture of DI water and 2-isopropanol for 10 min to ensure the removal of the polishing alumina powder. Then, the polished Au electrode was mounted on a rotator (RRDE-3A, ALS) and immersed into the electrolyte. Before starting the electrochemical experiment, each electrolyte was prebubbled by Ar for at least 20 min. Then, linear sweep voltammetry was conducted to measure HER kinetics on the Au surface (scan rate of 5 mV s^–1^ at 3000 rpm rotation speeds). For each measurement, *iR* correction was applied at 85% compensation of the uncompensated resistance values measured by electrochemical impedance spectroscopy.

### Preparation of Au Electrode for DEMS Measurement

The nanoporous PTFE membrane (20 nm of pore size, Cobetter Filtration Equipment) was used as both a pervaporation membrane for DEMS and a substrate for the Au electrocatalyst. Before Au deposition, the PTFE membrane was sonicated in ethanol for 30 min. A 400 nm thick Au thin film was deposited on the PTFE membrane for optimal product molecule detection^32^ using electron-beam physical vapor deposition (Creamet 450 e-beam). The polycrystalline structure of the deposited Au thin film was confirmed by using X-ray diffraction (XRD) (Figure S1).

### Electrochemical Flow Cell for *In Situ* DEMs

An *in situ* DEMS experiment was conducted using a two-compartment homemade three-electrode PEEK flow cell (design similar to previous literature^33^). The Au-deposited nanoporous PTFE membrane served as a working electrode, Pt mesh served as a counter electrode, and leakless Ag/AgCl (eDAQ) as a reference. The cation-exchanged Nafion membrane was used to separate the cathode and anode compartments.

Before each experiment, the cell was thoroughly cleaned by soaking in 0.05 M H_2_SO_4_ solution for 1 h, followed by boiling in DI water for 10 min, which was repeated two times. Each electrode chamber was pumped with electrolytes using a peristaltic pump (Baoding Shenchen Precision pump) at a flow rate of 60 mL min^–1^. The catholyte and anolyte were pumped from/into separated electrolyte reservoirs to prevent species crossover from the anode to the cathode during the experiments.

### *In Situ* DEMS Experiments

Electrochemical measurements were carried out using a Biologic VSP-300 potentiostat. The electrochemical surface area (ECSA) of the Au thin film working electrode is calculated by dividing the reduction charge of Au oxide during cyclic voltammetry in 0.05 M H_2_SO_4_ by the specific reduction charge of a Au oxide monolayer on polycrystalline Au (390 μC cm_Au_^–2^) (Figure S2).^44−49^ This value is used for calculating the ECSA-normalized current density. Before each experiment, the electrolytes were saturated by Ar or CO_2_ for at least 20 min prior being pumped into the flow cell, for HER or eCO_2_RR measurements, respectively. Next, the Au thin film electrode (on PTFE membrane) was conditioned in each electrolyte by conducting cyclic voltammetry between 0.4 and 1.7 V (vs RHE) at a scan rate of 50 mV s^–1^ for three cycles. Then, linear sweep voltammetry (LSV) was conducted at a scan rate of 5 mV s^–1^ for both HER and eCO_2_RR measurements. During LSV, mass spectra were acquired by an HPR40 mass spectrometer (Hiden Analytic) at 70 eV of electron energy and 500 μA of emission current. A secondary electron multiplier detector was used at a voltage of 1350 V.

For quantitative product analysis, a calibration of H_2_ and CO across the Au-deposited nanoporous PTFE membrane was conducted by applying a modified methodology from the literature.^33^ Briefly, H_2_ calibration was conducted *in situ* by directly comparing the HER current from LSV and H_2_ signal (*m*/*z* = 2) at each potential in the Ar-saturated KHCO_3_ electrolyte (Figure S3). Then, the HER current density was plotted against the *m*/*z* = 2 mass-ion signal, and using linear fitting, a H_2_ calibration was generated and later on used to convert the measured *m*/*z* = 2 mass-ion signal to HER current density (Figure S4). The accuracy of the H_2_ calibration was confirmed by observing an overlap between the HER current directly measured from the potentiostat and the HER current calculated from the DEMS *m*/*z* = 2 signal using a calibration curve (Figure S5). Next, CO signal calibration was conducted by measuring the *m*/*z* = 2, 28, and 44 mass-ion signals together. Using the H_2_ calibration results, the eCO_2_RR partial current was obtained by calculating the HER partial current first and then subtracting its contribution from the total current (Figure S6). Then, the produced net CO signal was calculated by subtracting the fragmental signal of CO_2_ (*m*/*z* = 44) to *m*/*z* = 28, which is denoted as *m*/*z*(CO) = 28. By plotting *m*/*z*(CO) = 28 against the eCO_2_RR partial current, a linear CO calibration line fitting was obtained (Figure S7), indicating that CO is the dominant eCO_2_RR product on the Au surface (in agreement with prior studies).^10,36^ Based on these calibrations, the Faradaic efficiency (FE) of eCO_2_RR was calculated (Figure S8).

## Computational Methods

### Molecular Dynamics

Classical molecular dynamics (MD) simulations were carried out using the LAMMPS program package (version 23 Jun 2022).^50^ System compositions and box dimensions are listed in Table S6. Force fields either based on OPLS-AA^51^ or designed to be compatible were used for K^+^,^52^ HCO_3_^–^,^53^ CF_3_COO^–^, C_2_H_5_COO^–^,^54^ and ClO_4_^–^.^55^ The SPC/E force field^56^ was used for water. The 12-6 Lennard-Jones force field parameters for face-centered cubic metals, as described by Heinz et al., were used to model non-Coulombic interactions with Au atoms.^57^ Lorentz–Berthelot mixing rules were applied for nonbonded interactions between different atom kinds.^58^ The cutoff distance for both Coulombic and Lennard-Jones interactions was set to 1.2 nm. The time step was set to 0.5 ns. Initial configurations for all simulations were created by random placement within the simulation box using PACKMOL.^59^ In order to avoid energetic hotspots, each simulation was preceded by an energy minimization and started with an *NVE* run for 0.03 ns with added velocity scaling corresponding to a temperature of 500 K. The Nosé–Hoover^60,61^ chain thermostat and barostat were employed to ensure an average pressure of 1.01325 bar in all *NpT* simulations and a temperature of 298.15 K in all *NpT* and *NVT* simulations.

Bulk simulations were performed for all investigated systems to determine their density. Periodic boundary conditions were applied in all directions. Following the initial energy minimization and *NVE* run, the systems were simulated in the *NpT* ensemble for 4 ns. The average density of the latter 2 ns was used to set up further simulations.

The recently implemented ELECTRODE package^62^ for constant potential simulations was used to simulate the solid–liquid interface at the electrode surface. Periodic boundary conditions were applied in the *x* and *y* directions. In the *z* direction, the liquid phase was confined by a 12 × 12 × 5 Au(111) surface slab on both sides of the simulations box. The atomic positions of the Au(111) electrodes were frozen during the entire simulation. The electrode distance was set to reproduce the bulk density determined in earlier simulations. During the initial equilibration, no constant potential was applied, and the atomic charge of all Au atoms was set to 0. Following the initial energy minimization and *NVE* run, the systems were equilibrated for 1 ns in the *NVT* ensemble. The constant potential method was then applied to calculate on-the-fly the atomic charges of the Au(111) electrodes so that a potential of 1 V was achieved. The systems were then further equilibrated for 8 ns. Finally, a production run of 8 ns was carried out under the same conditions. Trajectory analysis was performed using the postprocessing code TRAVIS.^63^ The relative coverage of the electrode surface by each species was estimated by weighting the particle density in the first adlayer with their van der Waals area according to Bondi’s list,^64^ assuming full coverage of the electrode surface.

To calculate the free energy profiles of the anions moving toward or away from the electrode at diluted conditions, another set of simulations was carried out employing the PLUMED package.^65^ Here, the systems were composed of water with a single ion pair placed inside. The liquid phase was confined in the *z* direction by a 10 × 10 × 5 Au(111) surface slab on either side of the simulation box. The cation’s position along the *z* axis was confined to a distance of 20 Å from the anode surface. The anion’s position along the *z* axis was controlled by the PLUMED package and varied between 2 and 20 Å distance from the cathode surface in steps of 0.5 Å. An independent simulation was carried out for each distance. Following the initial energy minimization and *NVE* run, the systems were equilibrated for 1 ns in the *NVT* ensemble. Afterward, a constant potential of 1 V was applied, and the systems were equilibrated for another 1 ns. Finally, a production run of 1 ns was performed under the same conditions.

## Supporting Information Available

*In situ* DEMS method characterization and calibration; MD simulation details; and supplementary discussion on different anion effects (PDF)
