# Supplementary material for: Anion Effect in Electrochemical CO2 Reduction: From Spectators to Orchestrators
Source: J Am Chem Soc. 2024 Oct 15;146(46):31768–77. doi: 10.1021/jacs.4c10661 (PMC11583205; doi:10.1021/jacs.4c10661)
Supplement: Supplementary file 1 — ja4c10661_si_001.pdf [file ja4c10661_si_001.pdf]

Supporting information

Anion Effect in Electrochemical CO_2 Reduction: From Spectators to Orchestrators

Ji Mun Yoo[a], Johannes Ingenmey[b], Mathieu Salanne[b,c], and Maria R. Lukatskaya[a,*]

[a]Electrochemical Energy Systems Laboratory, Department of Mechanical and Process Engineering, ETH Zurich, 8092 Zurich, Switzerland

[b]CNRS, Physicochimie des Électrolytes et Nanosystèmes Interfaciaux, Sorbonne Université, F-75005 Paris, France

[c]Institut Universitaire de France (IUF), 75231 Paris, France

*Correspondence: mlukatskaya@ethz.ch

The PDF includes:

Figure S1. *X-ray diffraction pattern of Au thin-films deposited on nanoporous PTFE membrane.* 3

Figure S2. *Cyclic voltammogram of Au thin-film electrode in Ar-saturated 0.05 M H_2SO_4 electrolyte for electrochemical surface calculation.* 4

Figure S3. in situ DEMS analysis of HER current density on Au electrode in Ar-saturated 0.1 M $KHCO_3$ electrolyte. 5

Figure S4. *H_2 calibration in three individual in situ DEMS experiments.* 6

Figure S5. *Comparison of HER current density between potentiostat measurement and DEMS mass-ion calculation result.* 7

Figure S6. *Deconvolution of total current density measured in CO_2-saturated 0.1 M $KHCO_3$ electrolyte into j_{H2} (HER) and j_{CO} (eCO_2RR) partial current.* 8

Figure S7. *CO calibration in CO_2-saturated 0.1 M $KHCO_3$.* 9

Figure S8. *Faradaic efficiency of eCO_2RR on Au electrode in CO_2-saturated 0.1 M $KHCO_3$.* 10

Figure S9. *Total current density of cathodic reaction on Au electrode in CO_2-saturated electrolytes.* 11

Figure S10. *eCO_2RR partial current density (j_{CO}) comparison on Standard Hydrogen Electrode (SHE) potential scale.* 12

Table S1. *pK_a or pK_b value of anions and their conjugate acids.* 13

Table S2. *pH value of 0.1 M potassium-based electrolytes when saturated with Ar and CO_2 gas.* 14

Figure S11. *HER activity of polycrystalline Au electrode in 8.4 mM CH_3COOH-added 0.1 M CH_3COOK (Ar-saturated, pH=5.8) (Scan rate = 5 mV s^{-1}).* 15

Table S3. *Comparison of electrochemical CO_2 reduction onset potential (at j_{CO} = -0.1 mA cm^2).* 16

Figure S12. *Total current density of cathodic reaction on Au electrode in CO_2-saturated electrolytes.* 17

Figure S13. *Comparison of DEMS mass signal for consumed CO_2 (m/z=44, hollow) and evolved CO (m/z=28, CO_2 fragmentation-corrected, solid).* 18

Figure S14. *DEMS m/z=44 mass signal for CO_2 molecule measured at OCV condition for different 0.1 M electrolytes.* 19

Figure S15. *Calculated bicarbonate concentration in CO_2-saturated 0.1 M electrolytes.* 20

Table S4. *pH value and calculated bicarbonate concentration in CO_2-saturated electrolytes.* 21

Figure S16. *Linear sweep voltammetry curve for j_{H2} measured in Ar-saturated electrolytes using rotating Au disk electrode.* 22

Figure S17. *Box visualization of molecular dynamics calculated for different electrolytes.* 23

Table S5. *Calculated surface coverage of water, potassium cation, and anion charged Au(111) surfaces.* 26

Figure S18. *Evolution of the negative electrode charge (for an applied potential of 1V) with respect to the simulation time.* 25

Figure S19. *Surface charge carried by the electrode atoms for an instantaneous configuration of the system.* 27

Figure S20. *MD-calculated particle density of bicarbonate, perchlorate, and propionate anions with the distance away from Au(111) surface.* 27

Figure S21. *Potential of mean force for anions on Au(111) electrode surface with different potential applied.* 28

Figure S22. *MD-generated $O_{H2O} – H_{H2O}$ radial distribution function in g(r) space for main intermolecular interactions depending on different anion types.* 29

Table S6. *Composition and final box dimensions of the simulated systems (d_{el}: the distance between electrode surfaces).* 30

Supplementary Discussion 31

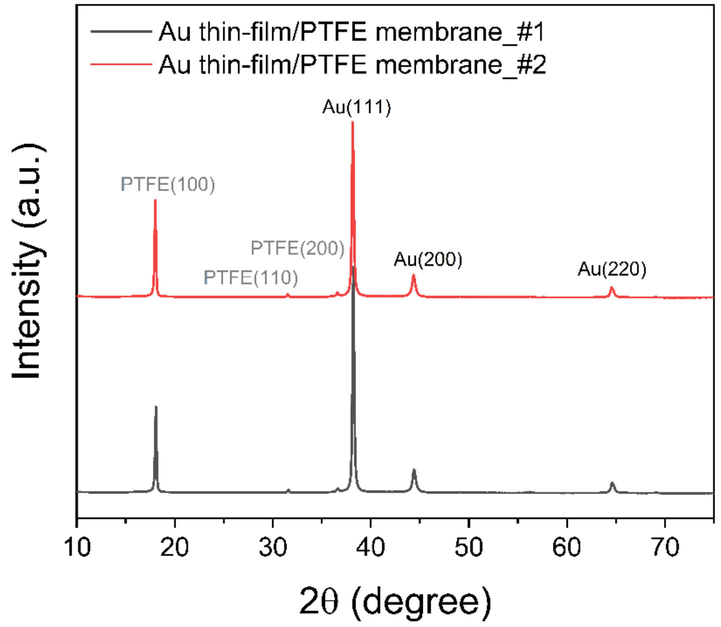

Figure S1. X-ray diffraction pattern of Au thin-films deposited on nanoporous PTFE membrane.

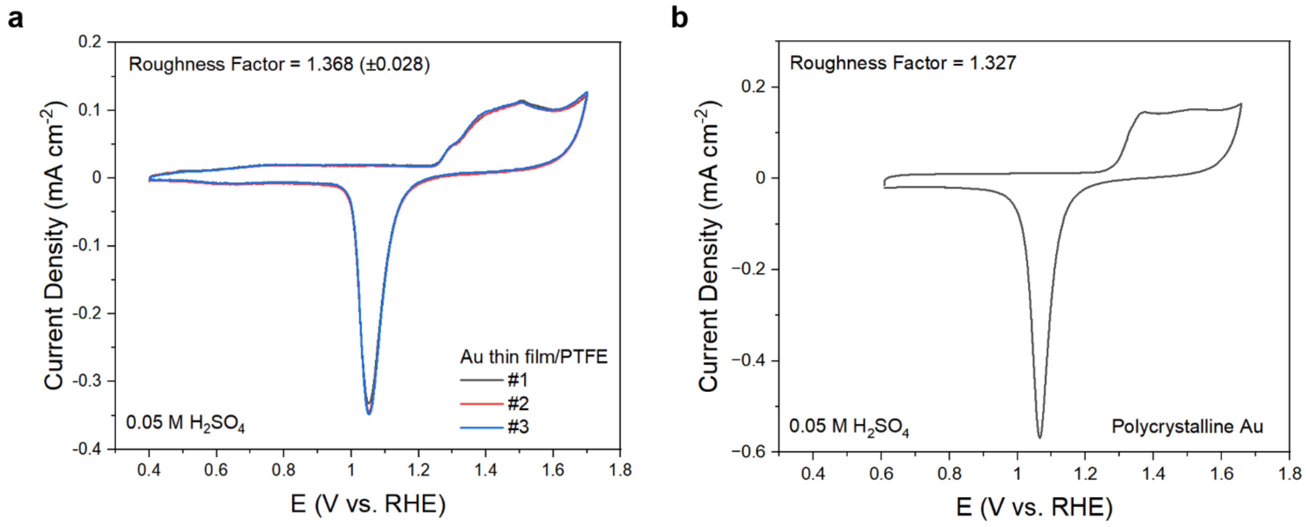

Figure S2. Cyclic voltammogram of (a) Au thin-film electrode on PTFE membrane and (b) polycrystalline Au in Ar-saturated 0.05 M H_2SO_4 electrolyte.

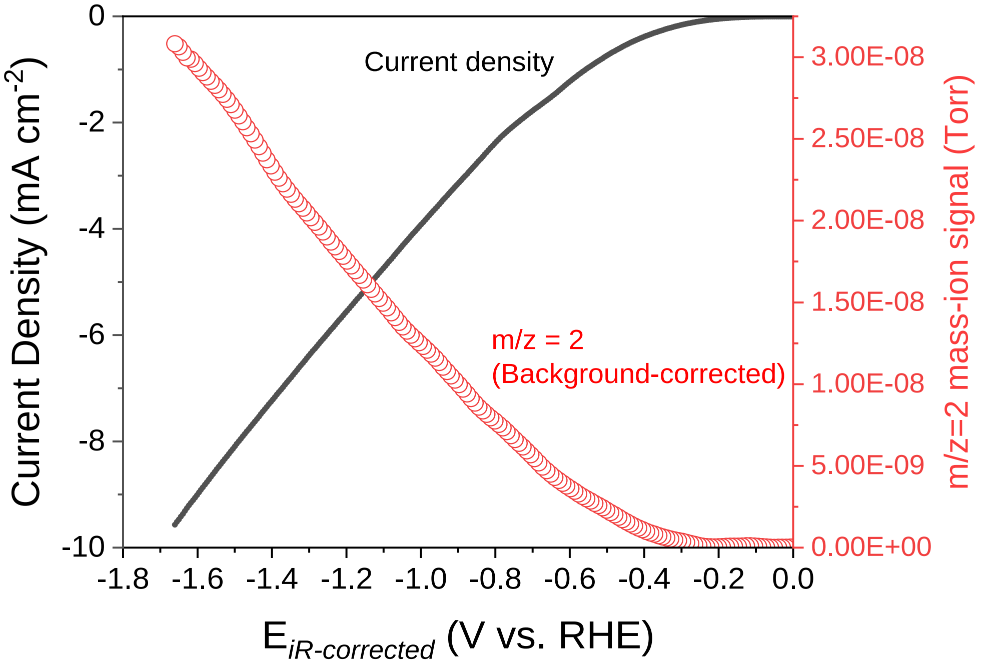

Figure S3. *in situ* DEMS analysis of HER current density on Au electrode in Ar-saturated 0.1 M $KHCO_3$ electrolyte: Electrochemical current density (black) and m/z=2 mass-ion signal (red).

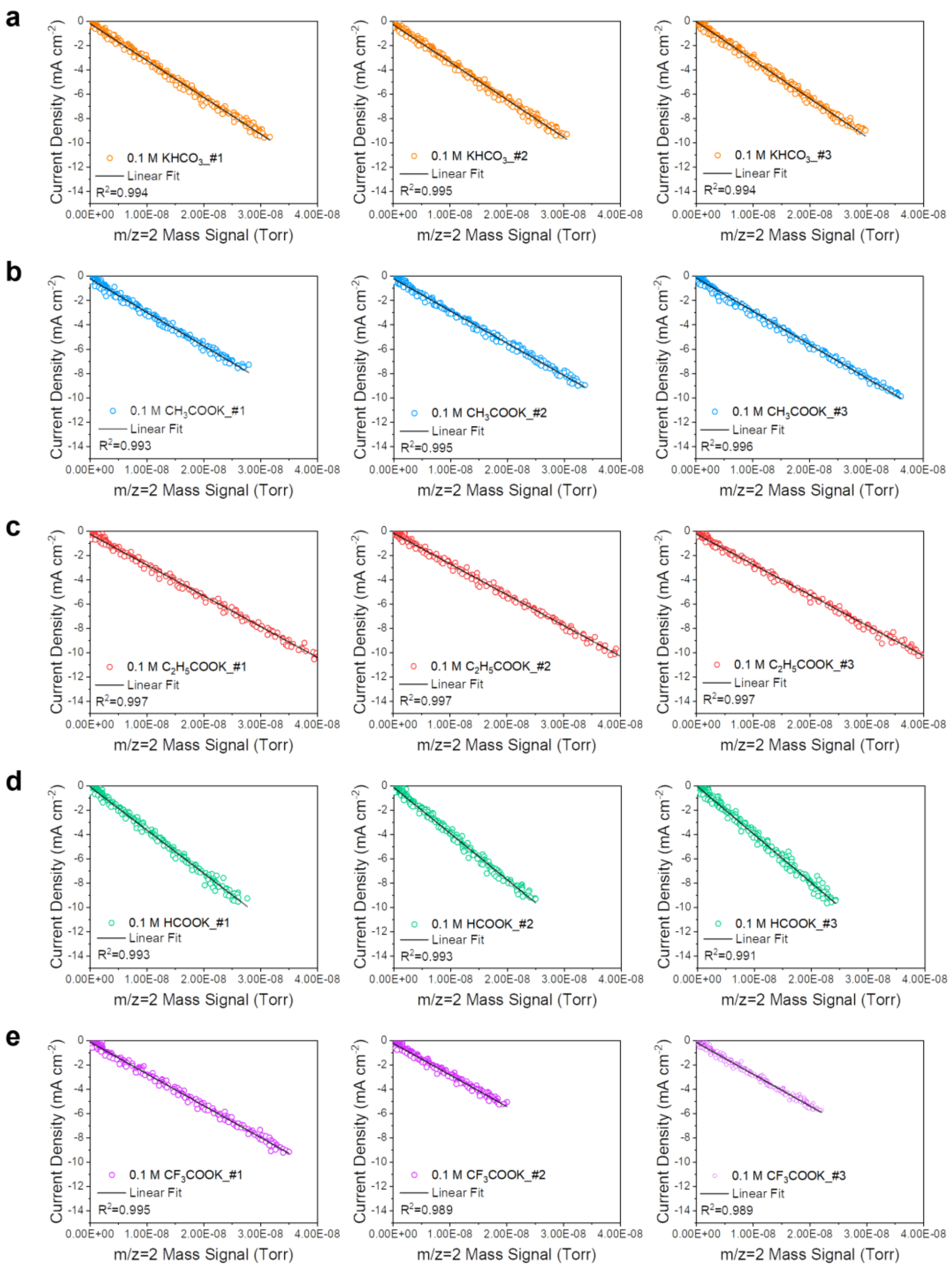

Figure S4. H_2 calibration in three individual *in situ* DEMS experiments by comparing HER current density and m/z=2 mass-ion signal in Ar-saturated electrolytes: (a) 0.1 M $KHCO_3$, (b) 0.1 M CH_3COOK, (c) 0.1 M C_2H_5COOK, (d) 0.1 M HCOOK, and (e) 0.1 M CF_3COOK.

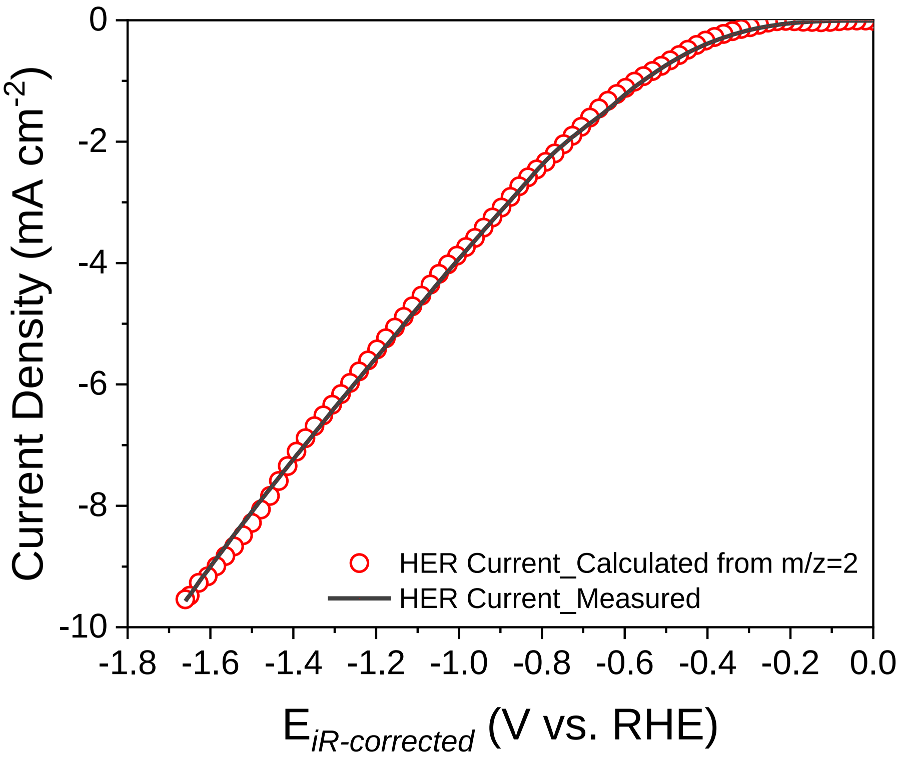

Figure S5. Comparison for Ar-saturated 0.1 M $KHCO_3$ electrolyte between HER current density directly measured from potentiostat and HER current density calculated from m/z=2 mass-ion DEMS signal by using H_2 calibration result.

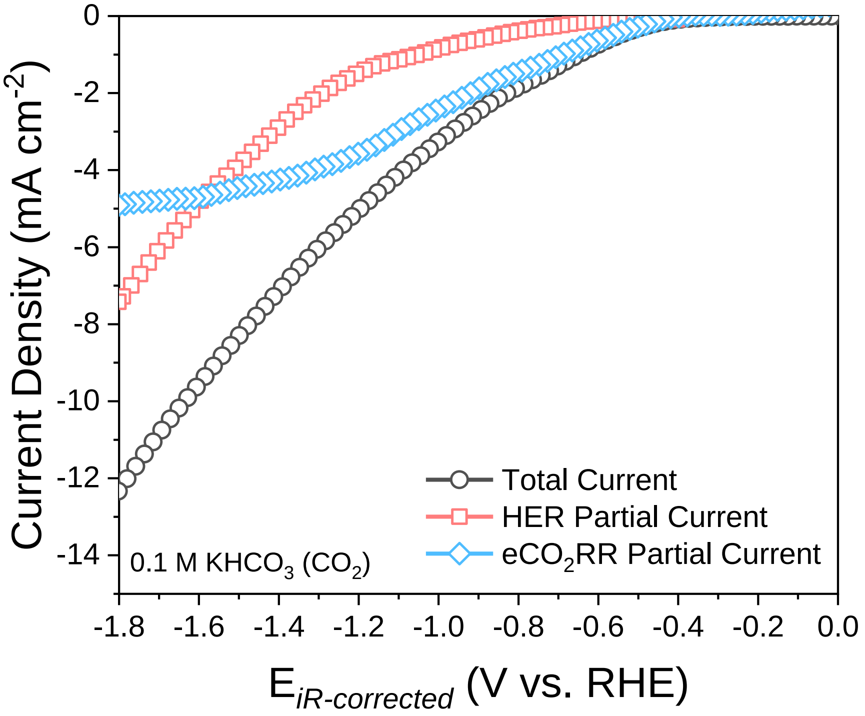

Figure S6. Deconvolution of total current density measured in CO_2-saturated 0.1 M $KHCO_3$ electrolyte into j_{H2} (HER) and j_{CO} (eCO_2RR) partial current.

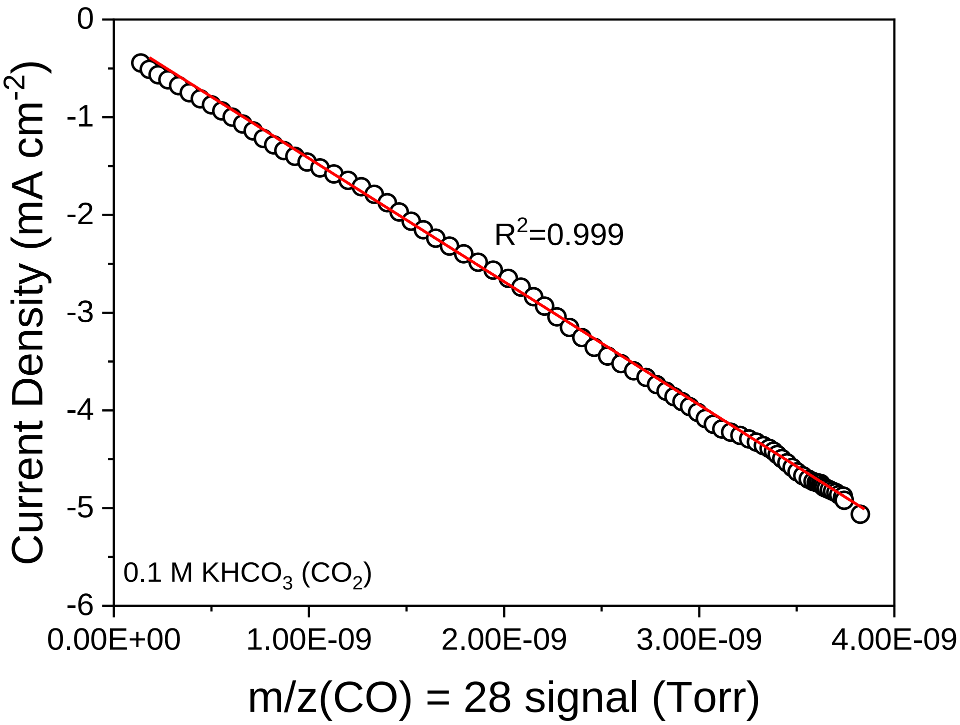

Figure S7. CO calibration in CO_2-saturated 0.1 M $KHCO_3$ by comparing j_{CO} and m/z=28 mass-ion signal after correction of CO_2 (m/z=44) fragmentation into m/z=28.

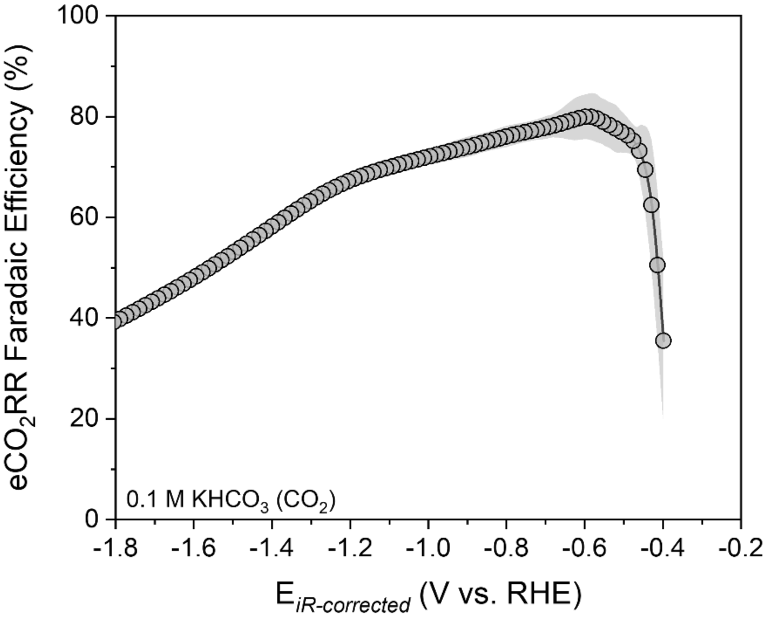

Figure S8. Faradaic efficiency of eCO_2RR on Au electrode in CO_2-saturated 0.1 M $KHCO_3$. Standard deviation results at each potential are calculated from three independent experiments and plotted as shade (grey).

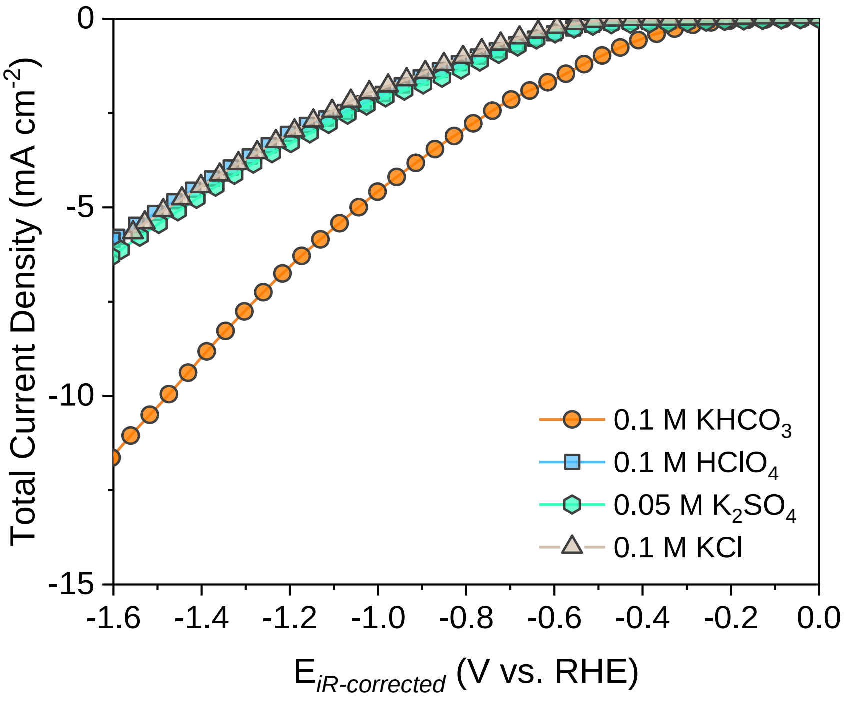

Figure S9. Total current density of cathodic reaction on Au electrode in CO_2-saturated 0.1 M $KHCO_3$, 0.1 M $KClO_4$, 0.05 M K_2SO_4, and 0.1 M KCl electrolytes measured by *in situ* DEMS cell (Scan rate: 5 mV s^{-1}, Flow rate: 60 mL min^{-1}).

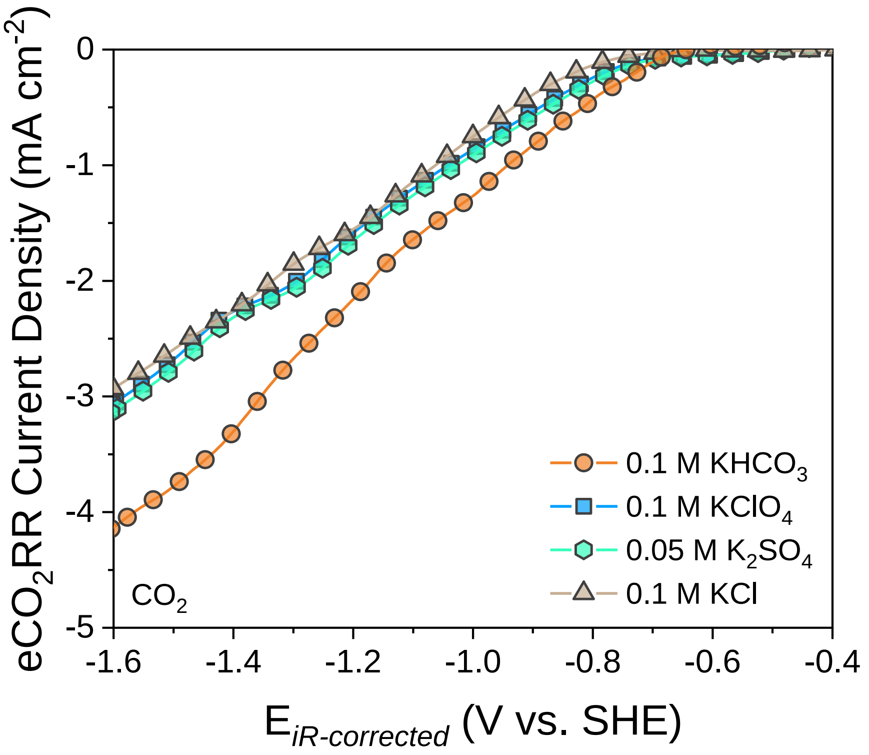

Figure S10. eCO_2RR partial current density (j_{CO}) comparison on Standard Hydrogen Electrode (SHE) potential scale between 0.1 M $KHCO_3$, 0.1 M $KClO_4$, 0.05 M K_2SO_4, and 0.1 M KCl electrolytes.

Table S1. pK_a or pK_b value of anions and their conjugate acids.

Anions	HCO_3^-	$CH_3CO_2^-$	$C_2H_5CO_2^-$	HCO_2^-	$CF_3CO_2^-$	ClO_4^-	SO_4^{2-}	Cl^-
pK_a	10.3	-	-	-	-	-	-	-
pK_b	7.65	9.24	9.12	10.26	13.77	>14	>14	>14
Conjugate Acid	H_2CO_3	CH_3COOH	C_2H_5COOH	HCOOH	CF_3COOH	$HClO_4$	HSO_4-	HCl
pK_a	6.35	4.76	4.88	3.74	0.23	-10.0	-2.0	-6.0

Table S2. pH value of 0.1 M potassium-based electrolytes when saturated with Ar and CO_2 gas.

$[K^+]$ = 0.1 M	HCO_3^-	$CH_3CO_2^-$	$C_2H_5CO_2^-$	HCO_2^-	$CF_3CO_2^-$	ClO_4^-	SO_4^{2-}	Cl^-
pH (Ar sat.)	8.4	7.4	7.7	6.8	6.7	6.7	6.7	6.7
pH (CO_2 sat.)	6.8	5.8	5.8	5.2	4.6	4.0	4.0	4.0

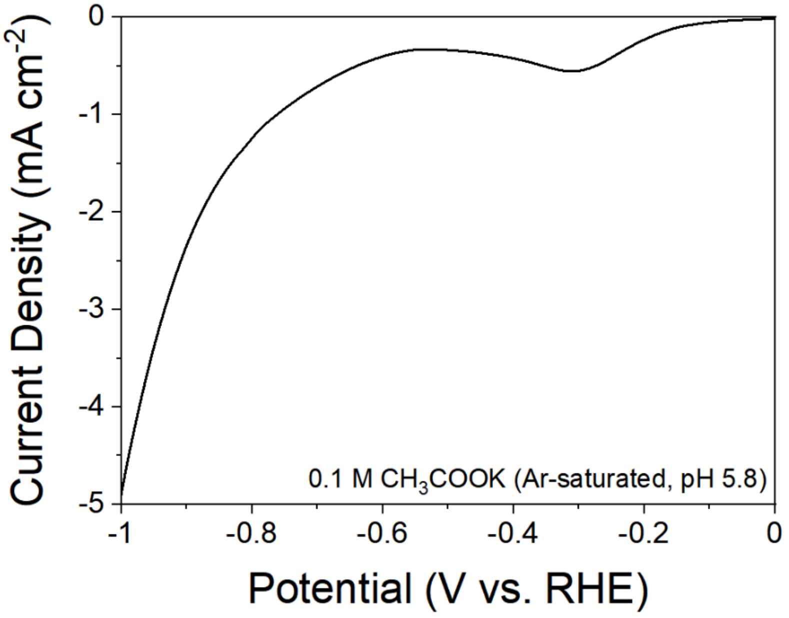

Figure S11. HER activity of polycrystalline Au electrode in 8.4 mM CH_3COOH-added 0.1 M CH_3COOK (Ar-saturated, pH=5.8) (Scan rate = 5 mV s^{-1}).

Table S3. Comparison of electrochemical CO_2 reduction onset potential (at j_{CO} = -0.1 mA cm^2).

$[K^+]$ = 0.1 M	HCO_3^-	$CH_3CO_2^-$	$C_2H_5CO_2^-$	HCO_2^-	$CF_3CO_2^-$	ClO_4^-	SO_4^{2-}	Cl^-
$E_{onset,\ eCO2RR}$ (V vs. RHE)	-0.30	-0.23	-0.21	-0.39	-0.44	-0.49	-0.48	-0.52

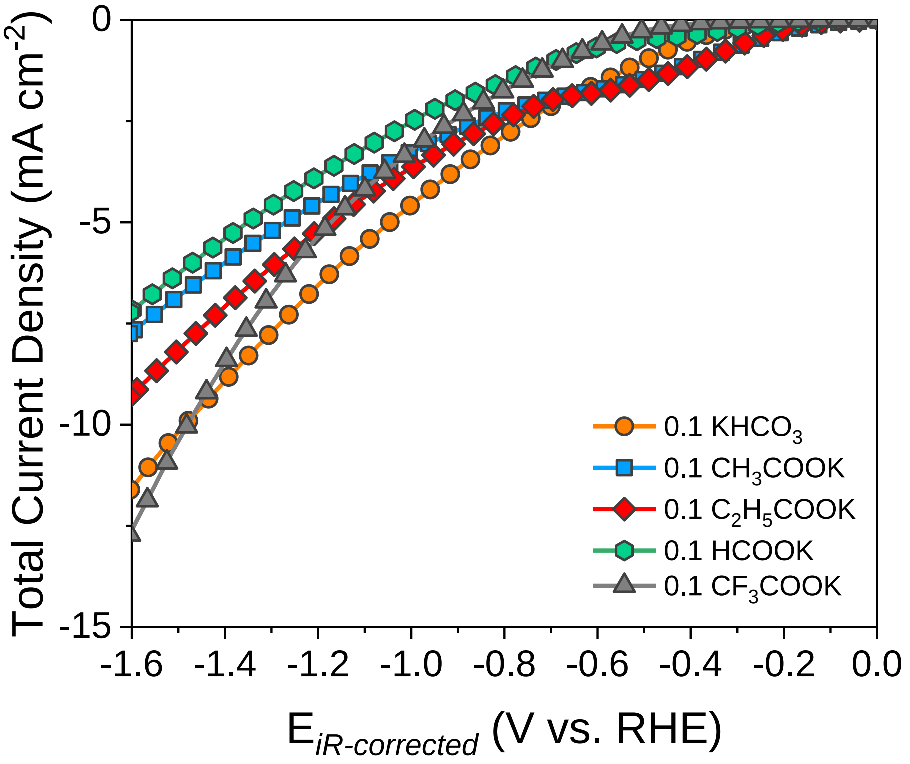

Figure S12. Total current density of cathodic reaction on Au electrode in CO_2-saturated 0.1 M $KHCO_3$, 0.1 M CH_3COOK, 0.1 M C_2H_5COOK, 0.1 M HCOOK, and 0.1 M CF_3COOK electrolytes measured by *in situ* DEMS cell (Scan rate: 5 mV s^{-1}, Flow rate: 60 mL min^{-1}).

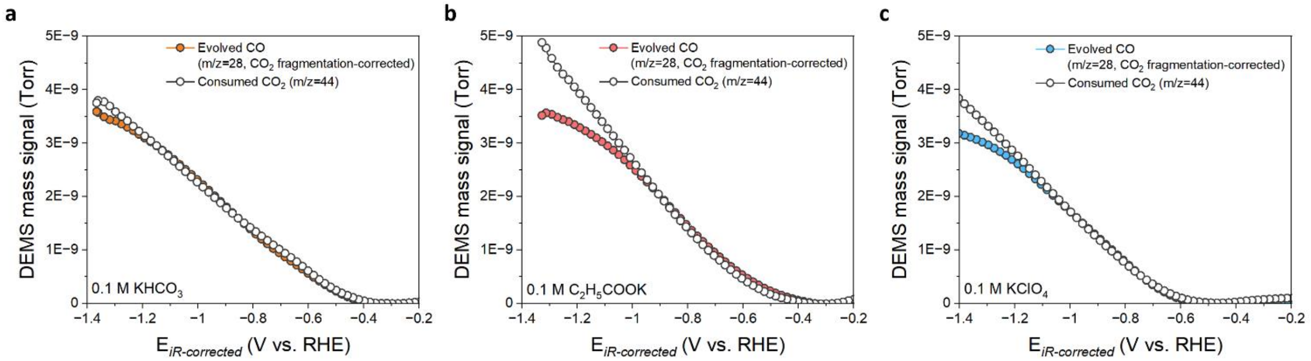

Figure S13. Comparison of DEMS mass signal for potential-dependent consumed CO_2 (m/z=44, hollow) and evolved CO (m/z=28, CO_2 fragmentation-corrected, solid) for (a) 0.1 M $KHCO_3$, (b) 0.1 M C_2H_5COOK, and (c) 0.1 M $KClO_4$. Consumed CO_2 mass signal is determined by difference in m/z=44 mass signal between each potential and open circuit potential (i.e. baseline). Evolved CO mass signal is calculated by subtracting partial fragmentation signal of CO_2 (m/z=44) from total m/z=28 mass signal.

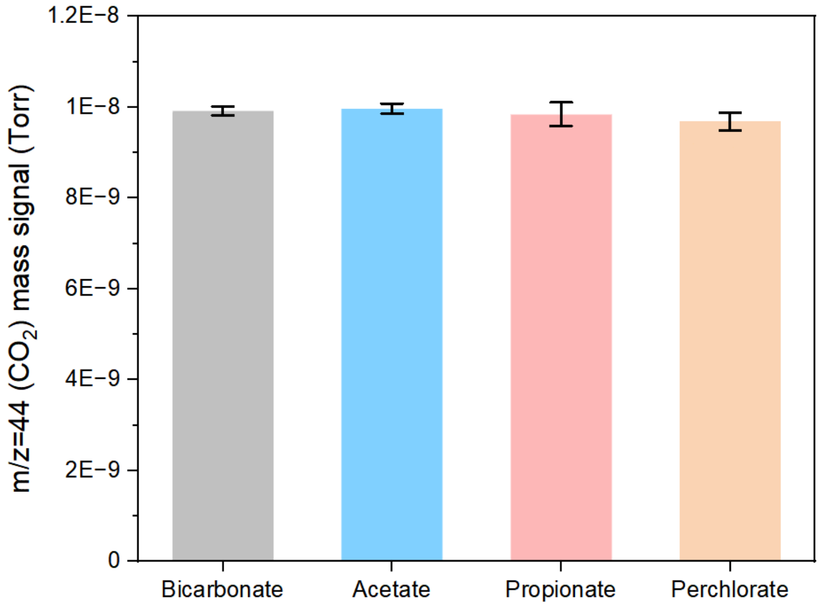

Figure S14. DEMS m/z=44 mass signal for CO_2 molecule measured at OCV condition for different 0.1 M electrolytes.

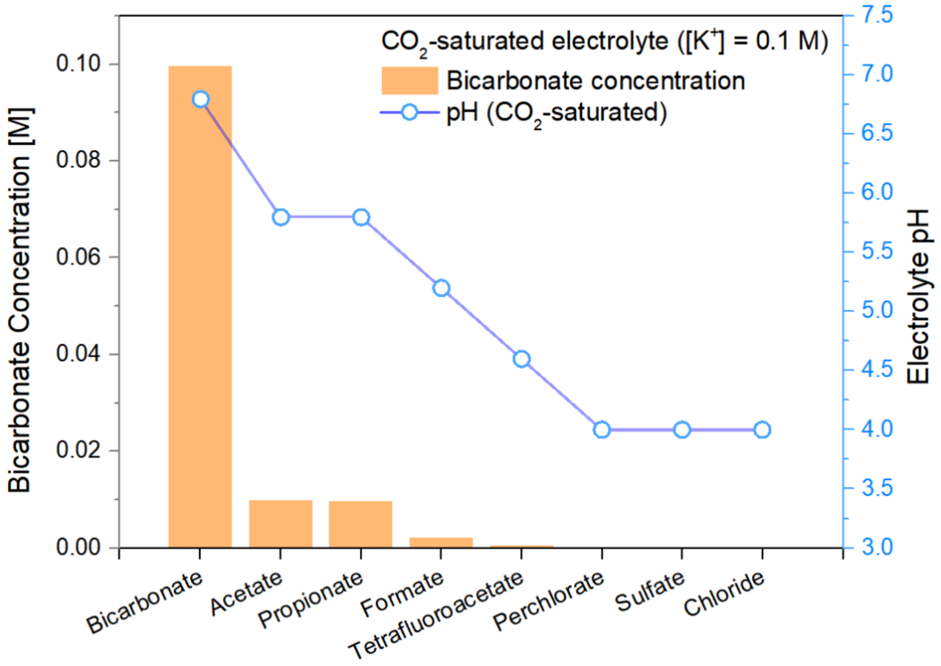

Figure S15. Calculated bicarbonate concentration in CO_2-saturated 0.1 M electrolytes.

Table S4. pH value and calculated bicarbonate concentration in CO_2-saturated electrolytes

**CO_2-saturated Electrolyte**	**0.1 M $KHCO_3$**	**0.1 M CH_3COOK**	**0.1 M C_2H_5COOK**	**0.1 M HCOOK**	**0.1 M CF_3COOK**	**0.1 M $KClO_4$**	**0.05 M K_2SO_4**	**0.1 M KCl**
pH	6.8	5.8	5.8	5.2	4.6	4.0	4.0	4.0
$[HCO_3^-]$ [M]	0.1	9.97×10^{-3}	9.74×10^{-3}	2.28×10^{-3}	0.57×10^{-3}	0.14×10^{-3}	0.14×10^{-3}	0.14×10^{-3}
HCO_3^- ratio (%)	100	9.97	9.74	2.28	0.57	0.14	0.14	0.14

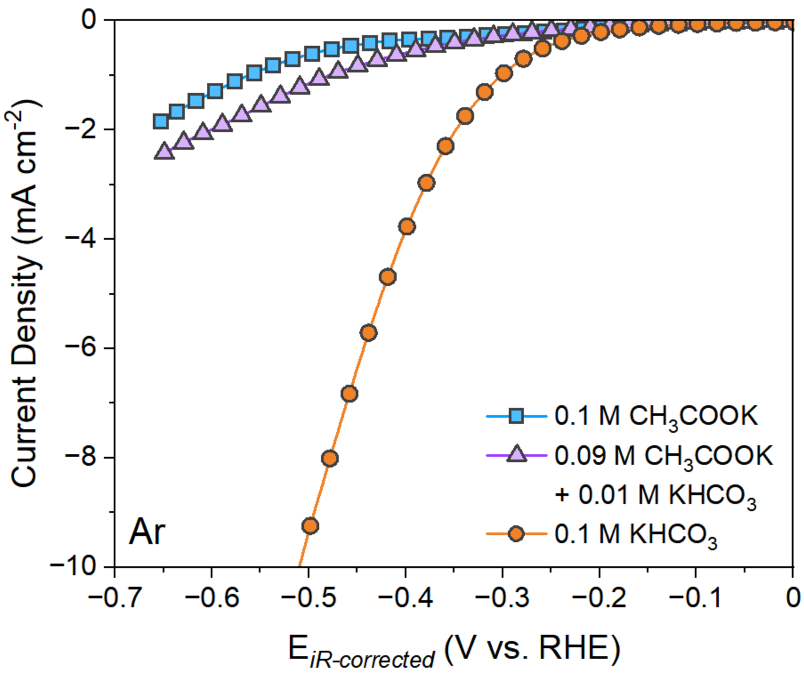

Figure S16. Linear sweep voltammetry curve for j_{H2} measured in Ar-saturated electrolytes using rotating Au disk electrode (Scan rate of 10 mV s^{-1}, rotation speed of 3000 rpm).

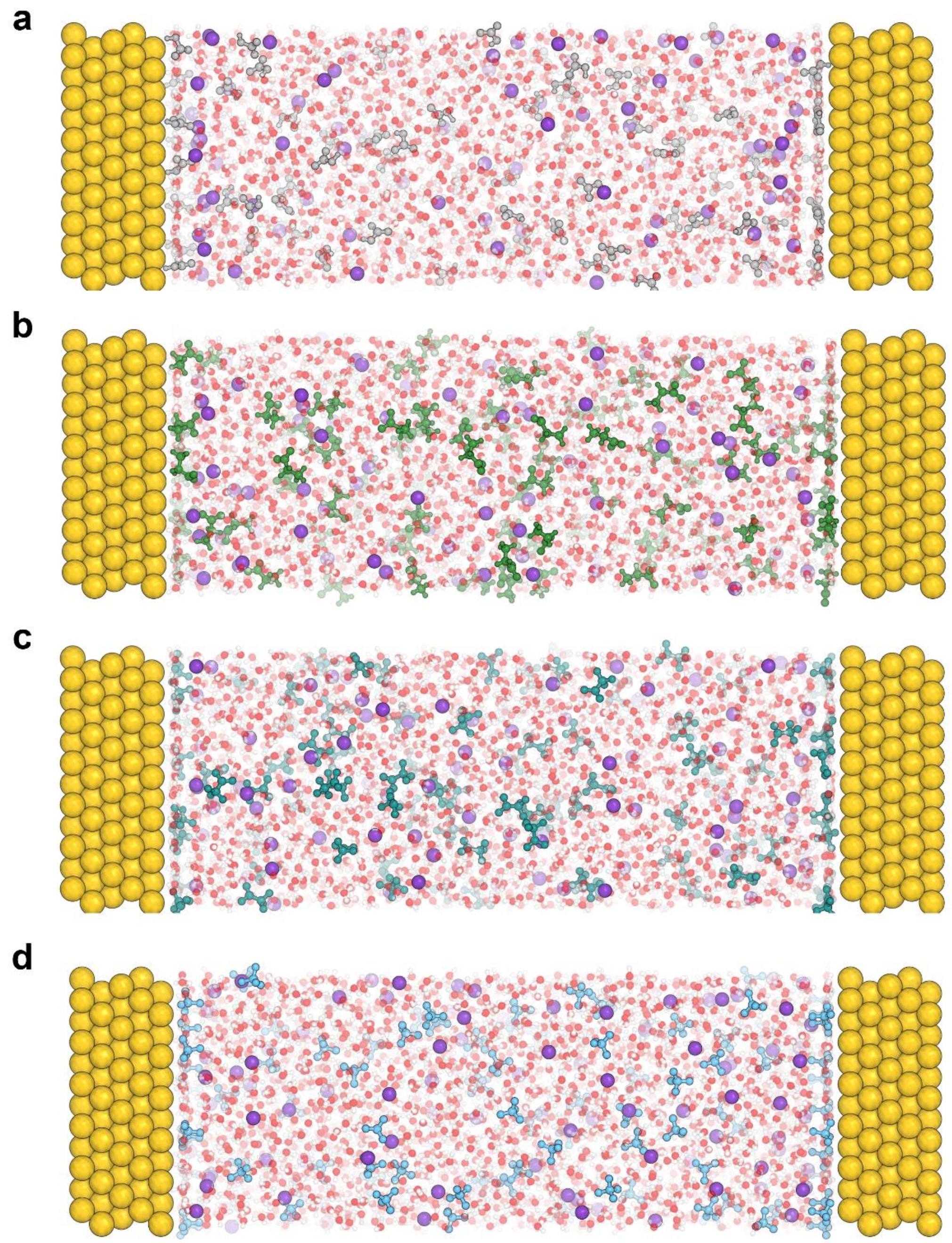

Figure S17. Box visualization of molecular dynamics calculated for (a) bicarbonate (grey), (b) propionate (green), (c) trifluoroacetate (dark blue), and (d) perchlorate (bright blue).

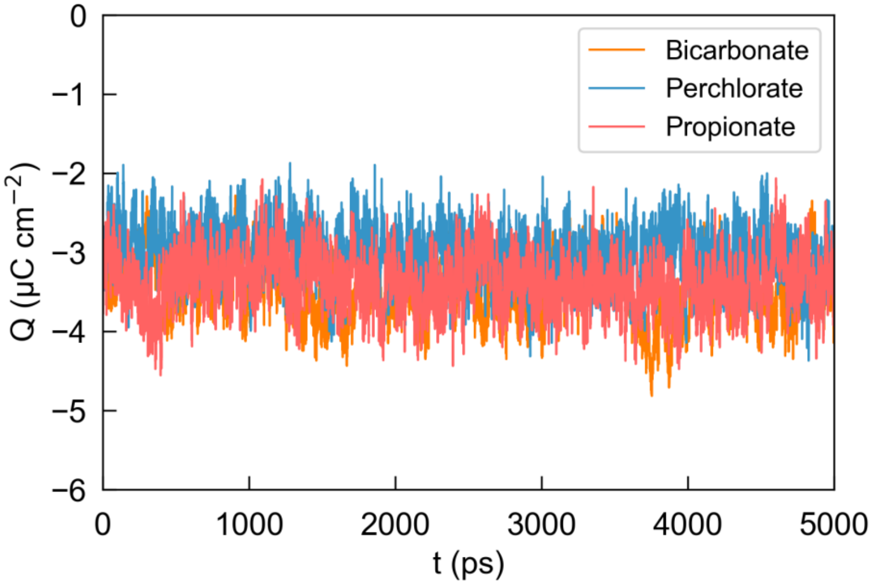

Figure S18. Evolution of the negative electrode charge (for an applied potential of 1V) with respect to the simulation time for the bicarbonate, perchlorate and propionate anions.

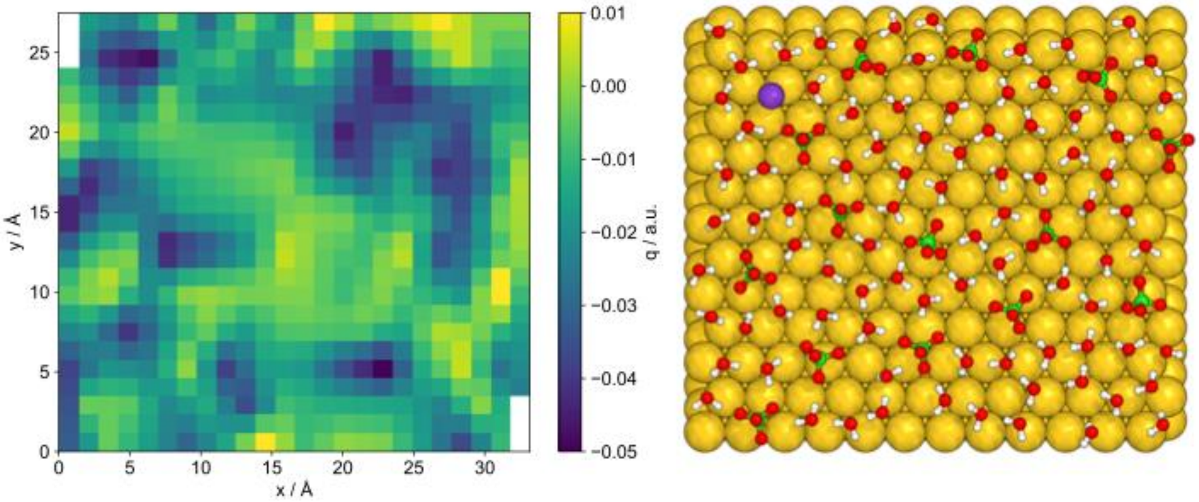

Figure S19. Left: Surface charge carried by the electrode atoms for an instantaneous configuration of the system. Right: positions of the adsorbed atoms for the same configuration (yellow: Au, red: O, white: H, violet: K, green: Cl)

Table S5. Calculated surface coverage of water, potassium cation, and anion charged Au(111) surfaces.

Cathode (negatively charged Au(111) surface)

Species	$KHCO_3$	C_2H_5COOK	CF_3COOK	$KClO_4$
H_2O	80.87%	78.13%	76.81%	73.04%
K^+	0.21%	0.30%	0.29%	1.74%
Anion	18.91%	21.57%	22.9%	25.23%

Anode (positively charged Au(111) surface)

Species	$KHCO_3$	C_2H_5COOK	CF_3COOK	$KClO_4$
H_2O	78.65%	76.44%	74.17%	68.57%
K^+	0.00%	0.01%	0.03%	0.66%
Anion	21.35%	23.55%	25.80%	30.77%

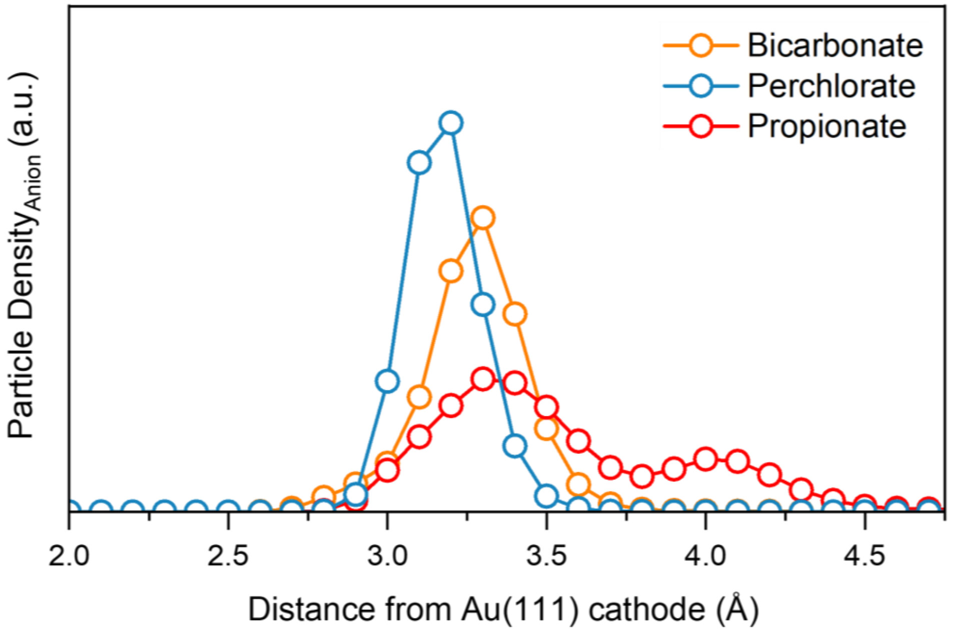

Figure S20. MD-calculated particle density of bicarbonate, perchlorate, and propionate anions with the distance away from Au(111) surface for the equilibrium simulation at 0 V.

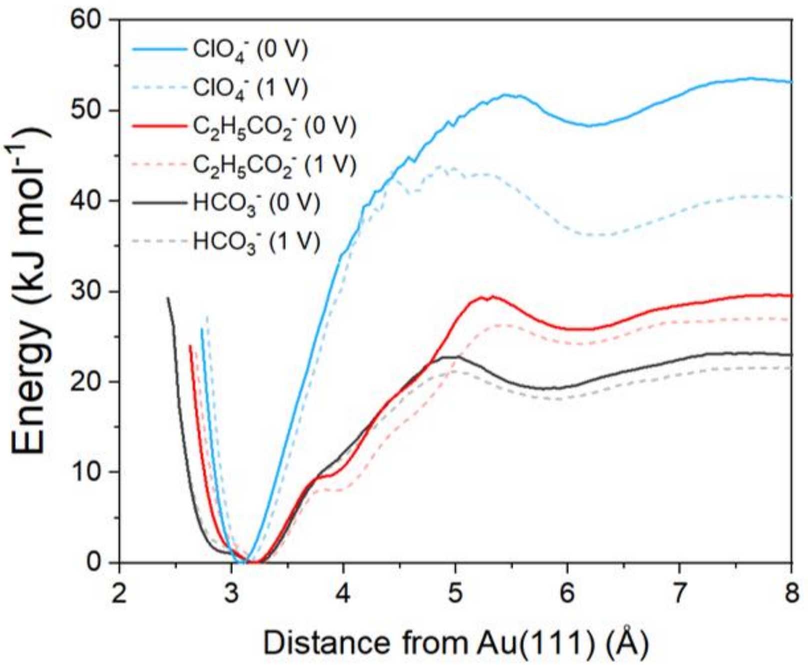

Figure S21. Potential of mean force for anions on Au(111) cathode surface with different potential applied: 0 V (solid) and 1 V (dashed).

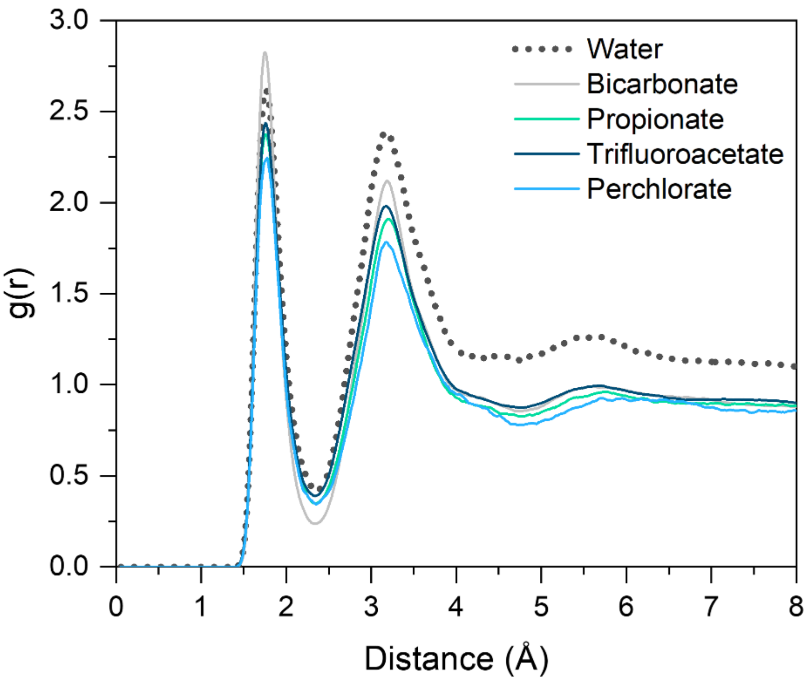

Figure S22. MD-generated O_{H2O} – H_{H2O} radial distribution function in g(r) space for main intermolecular interactions depending on different anion types.

Table S6. Composition and final box dimensions of the simulated systems (d_{el}: the distance between electrode surfaces).

Bulk	HCO_3^-	$C_2H_5CO_2^-$	ClO_4^-	$CF_3CO_2^-$
H_2O	2400	2400	2400	2400
K^+	100	100	100	100
A^-	100	100	100	100
a / Å	43.0881	43.8004	43.6255	43.9502
ρ / g/cm³	1.1053	1.0761	1.1418	1.1432

Coverage	HCO_3^-	$C_2H_5CO_2^-$	ClO_4^-	$CF_3CO_2^-$
H_2O	2400	2400	2400	2400
K^+	100	100	100	100
A^-	100	100	100	100
Au	1440	1440	1440	1440
a / Å	34.6199	34.6199	34.6199	34.6199
b / Å	29.9818	29.9818	29.9818	29.9818
c / Å	100.9163	102.3009	109.3147	102.4095
d_{el} / Å	82.0716	83.4561	82.4904	83.5647

PMF	HCO_3^-	$C_2H_5CO_2^-$	ClO_4^-	$CF_3CO_2^-$
H_2O	1200	1200	1200	1200
K^+	1	1	1	1
A^-	1	1	1	1
Au	1000	1000	1000	1000
a / Å	28.8500	28.8500	28.8500	28.8500
b / Å	24.9848	24.9848	24.9848	24.9848
c / Å	70.8444	70.8444	70.8444	70.8444
d_{el} / Å	51.9996	51.9996	51.9996	51.9996

Supplementary Discussion: Bicarbonate formation in CO_2 saturated electrolytes

The amount of bicarbonate in each electrolyte can be calculated from both CO_2 bulk concentration and solution pH. First, according to Henry's law (**Equation S1**), CO_2 solubility in each electrolyte under 1 atm of CO_2 in headspace is estimated to be 33 mM for all electrolytes (König *et al. iScience* 2019, 19, 135-160), assuming negligible salting-out effect at dilute 0.1 M electrolyte concentration.

$$C_{CO_2} = k_H \times p_{CO_2} \qquad \text{(Equation S1)}$$

(C_{CO_2}: dissolved CO_2 concentration; k_H: Henry's law constant; p_{CO_2}: CO_2 partial pressure. $k_H = 3.3 \times 10^{-2}\ mol/L \cdot atm$)

This assumption can be confirmed by the close similarity in CO_2 mass signal (m/z=44) from DEMS measurement at open circuit potential (**Figure S14**).

Next, the equilibrium bicarbonate concentration in each electrolyte can be calculated using Henderson-Hasselbalch equation (**Equation S2**).

$$pH = pK_{a,H_2CO_3} + log_{10}\left(\frac{[HCO_3^-]}{[H_2CO_3]}\right) \qquad \text{(Equation S2)}$$

Since pK_{a,H_2CO_3} value of 6.35, expected bicarbonate concentration in perchlorate, chloride, and sulfate electrolyte (Note: electrolyte pH of 4 with CO_2 saturation) is below 0.2 mM which is less than 0.2% compared to 0.1 M of those anions. For carboxylate, bicarbonate concentration ranges from 10 mM to 0.5 mM from acetate to trifluoroacetate, respectively, because of different electrolyte pH after CO_2 saturation (Table S2). The result is summarized in **Figure S15** and **Table S4**.

As seen in Figure S15, bicarbonate formation from CO_2 hydration is largely limited for formate, tetrafluoroacetate, perchlorate, sulfate, and chloride due to its low pH value (**Table S3 and S4**). Therefore, potential contribution of bicarbonate for HER in those electrolytes should be very limited.

For acetate and propionate, bicarbonate concentration reaches around 10 mM, which could influence HER current. We also observe that the HER partial currents for acetate and propionate (**Figure 2b**), which are higher than for other anions (except bicarbonate) and are characterized by an early HER onset potential of E = -0.1 V (vs. RHE).

To determine whether the increased HER activity can be attributed to the presence of equilibrium bicarbonate amounts in CO_2 saturated solution of acetate (or propionate), we performed additional RDE studies of HER activity in 0.01 M $KHCO_3$ + 0.09 M CH_3COOK electrolyte (**Figure S16**) and compared to HER currents in 0.1 M CH_3COOK and 0.1 M $KHCO_3$ under Ar-saturated condition (without CO_2).

As can be seen from Figure S16, HER activity is slightly increased in 0.01 M $KHCO_3$ + 0.09 M CH_3COOK electrolyte compared to 0.1 M CH_3COOK. However, this increase is only marginal, especially when considering large HER activity difference between pure 0.1 M $KHCO_3$ and 0.1 M CH_3COOK.

Therefore, it can be concluded that 10 mM bicarbonate presence in 0.1 M CH_3COOK does not significantly contribute to overall HER activity.
